# smartPARE: An R Package for Efficient Identification of True mRNA Cleavage Sites

**DOI:** 10.3390/ijms22084267

**Published:** 2021-04-20

**Authors:** Kristian Persson Hodén, Xinyi Hu, German Martinez, Christina Dixelius

**Affiliations:** The Swedish University of Agricultural Sciences, Department of Plant Biology, Uppsala BioCenter, Linnean Center for Plant Biology, P.O. Box 7080, S-75007 Uppsala, Sweden; kristian.persson.hoden@slu.se (K.P.H.); xinyi.hu@slu.se (X.H.); german.martinez.arias@slu.se (G.M.)

**Keywords:** cleavage sites, *Phytophthora infestans*, potato, small RNA, *Solanum tuberosum*

## Abstract

Degradome sequencing is commonly used to generate high-throughput information on mRNA cleavage sites mediated by small RNAs (sRNA). In our datasets of potato (*Solanum tuberosum*, St) and *Phytophthora infestans* (Pi), initial predictions generated high numbers of cleavage site predictions, which highlighted the need of improved analytic tools. Here, we present an R package based on a deep learning convolutional neural network (CNN) in a machine learning environment to optimize discrimination of false from true cleavage sites. When applying smartPARE to our datasets on potato during the infection process by the late blight pathogen, 7.3% of all cleavage windows represented true cleavages distributed on 214 sites in *P. infestans* and 444 sites in potato. The sRNA landscape of the two organisms is complex with uneven sRNA production and cleavage regions widespread in the two genomes. Multiple targets and several cases of complex regulatory cascades, particularly in potato, was revealed. We conclude that our new analytic approach is useful for anyone working on complex biological systems and with the interest of identifying cleavage sites particularly inferred by sRNA classes beyond miRNAs.

## 1. Introduction

RNA interference is a major components of eukaryotic gene regulation machinery at the posttranscriptional and transcriptional levels. In *Arabidopsis thaliana*, this mechanism, termed RNA silencing, is involved in the regulation of numerous processes, including fertilization, development, and the response to abiotic and biotic stresses. RNA silencing is associated with the activity of small RNAs (sRNAs) for which several classes have been categorized according to differences in their RNA template and role [1]. The first categorization layer is defined by the precursor RNA of sRNAs which can be a double-stranded RNA or single-stranded hairpin RNAs (hpRNAs). These two main categories are divided into several sub-classes and may cause overlapping cascades of various types of sRNAs. MicroRNA (miRNA) belong to the hpRNA category and are important regulations of many functions. They are processed from an RNA polymerase II-transcribed primary miRNA (pri-miRNA). This pri-miRNA is recognized by DICER-LIKE 1 (DCL1) endonuclease that produces a precursor RNA (pre-miRNA), which is further processed by the same endonuclease to produce a miRNA/miRNA star (miRNA *) duplex. The duplex disassembles when the mature miRNA binds to an ARGONAUTE (AGO) protein [2]. The miRNA/AGO complex directs post-transcriptional regulation of mRNAs by the complementarity between the miRNA and target RNA where 2–7 nucleotides called seed region are needed. This interaction directs the cleavage of target mRNA by endonuclease activity by AGO. Alternatively, the miRNA/AGO–mRNA association causes translational repression. Upon miRNA cleavage, secondary siRNAs can be triggered initiating cascades of events. For example, secondary siRNA biogenesis may result in “phasing” resulting in siRNA populations with defined 21–22 nt intervals [3]. Alternatively, secondary siRNAs may target mRNA distinct from their precursor RNA, leading to accumulation of *trans*-acting siRNAs or tasiRNAs [4]. Confirmation of miRNA/siRNA activity and the identification of their whole array of target mRNAs is challenging at the experimental level. Several strategies have been developed in plants, which consider the singular fingerprint of plant miRNA activity, a cleavage site on the target mRNAs located between nucleotides 9–11 of the miRNA complementary sequence [5,6]. Techniques such as degradome sequencing and parallel analysis of RNA ends (PARE) use the molecular characteristics of RNAs targeted for degradation (presence of a 5′ monophosphate in mRNAs instead of the regular CAP extreme, similar to 5′ RACE) to ligate an adaptor that is used to produce high-throughput sequencing-compatible amplicons [5,6]. Although these methods have produced data that have helped to identify the targets and activity of miRNAs/siRNAs, the identification of true events of sRNA-mediated cleavage remains a challenging bioinformatic analysis, with the identification of a high number of false positives.

The potato (*Solanum tuberosum,* St) genome has a haploid genome size of about 840 Mb [7] and contains about 18% of transposable elements (TEs) where long terminal repeat (LTR) type retrotransposons dominate [8]. Regarding the components of its RNA silencing machinery, 14 Argonautes (AGO), seven Dicer-like (DCL), and six RNA-dependent RNA polymerases (RDR) are present in the genome [9,10]. *AGO1* and *AGO10* are duplicated, three paralogs of *AGO4* are present, and orthologs of *AGO8* and *AGO9* are missing. *AGO15* is found in some *Solanaceae* species including potato and is elevated during infection by *Phytophthora infestans* (Pi). Additionally, four *DCL2*s and duplicates of *RDR1* and *RDR6*, and three *RDR3* [10] complete this mechanism. Until now, their different involvements upon stress are unknown.

The late blight disease is a major constrain worldwide incited by the oomycete *P. infestans*. This pathogen is well known to rapidly overcome control measures involving resistance breeding and fungicide treatments [11]. *P. infestans* has a large genome (240 Mb) that encodes close to 1000 effector genes harboring specific amino acid motifs such as RXLR effector proteins and the more sequence diverse crinkling and necrosis (CRN) protein family, all with potential capacity to support the infection process [12]. These effector genes are located in genomic regions rich in transposable elements (TEs). In contrast to plants, *P. infestans* encodes a moderate number of core components for functional RNA interference pathways: two Dicer-like enzymes, five Agos, and one Rdr [13]. Additionally, *P. infestans* lacks silencing-related proteins such as the cytosine methyltransferase HUA Enhancer 1 (HEN1), additional RNA polymerases such as RNA polymerase IV or V, class 2 ribonucleases III such Drosha, and 3′–5′ exoribonucleases such as ERI. In line with a reduced RNA silencing machinery, only one miRNA (miR8788) has been found in its genome [14] compared to plants that encode hundreds of miRNAs. Additional sRNA sequencing of *P. infestans* has revealed not only 21 nt sRNAs but also 25/26, 31/32, 35, and 40 nt size classes [14,15]. Sources for these sRNAs are TEs, followed by CRN and RXLR effectors. In addition, 19–40 nt tRNA fragments (tRFs) are produced, particularly pronounced during host infection [16]. When analyzing sRNAs loaded into PiAgo1, sRNAs from TEs and protein-coding genes were found together with miR8788 [17]. This suggests that these sRNAs can have a biological role, either in the pathogen or in the interaction with its host.

Small RNA-associated events during attack of plant disease inciting organisms, such as viruses, have been extensively studied [18,19]. Regarding eukaryotic pathogens, the first applicable approach of RNA interference was the design of transgenic plants with constructs that could target invading pathogens and thereby reduce disease reviewed by [20,21]. The ability to silence effector genes in *P. infestans* via host-inducing gene silencing is demonstrated [22], and the *P. infestans* encoded miRNA promotes disease in potato most likely via host translocation [23].

Here, we explored the genome-wide sRNA-mediated regulation of mRNA targets in detail using degradome sequencing. To overcome the high frequency of false-positive cleavage sites, we developed smartPARE, an advanced complement to earlier degradome analytic tools based on a deep learning convolutional neural network (CNN) in Keras [24]. As a proof of concept to our methodology, we first validated the smartPARE tool by analyzing known miRNA-mediated mRNA cleavage sites in Arabidopsis. Next, smartPARE was applied on 10 different sRNA datasets to infer cleavages in both potato and *P. infestans* during their interaction. We found 4073 cleavage sites in the plant host and 702 in *P. infestans.* New information of dual functionality of *P. infestans* effectors and induction of defense-related cascades in potato form an important knowledge base for deepening our understanding of this complex interaction.

## 2. Results

### 2.1. Overview of Data Processing

To uncover the regulatory potential of sRNAs during *P. infestans* infection of potato, we included data from degradome and sRNA sequencing from three different material groups: transgenic potato *StAGO1a-GFP* (StAGO1a dataset), transgenic *P. infestans PiAgo1-GFP* (PiAgo1 dataset) in various combinations, and additional materials for comparisons, here called background (BG dataset). All four datasets contain different subsets, as specified in Table S1. Henceforth, different sRNA classes are specified when distinguished in numbers or when involved in specific events. Quality control, filtering, and separation of sRNAs aligning to the genomes of potato or *P. infestans* resulted in five degradome and 10 sRNA datasets for further analysis. The sequence data were handled in the following workflow: data pre-processing, cleavage prediction, true cleavage identification, and dataset comparisons. Raw data were wrangled (converted between different data formats), quality-controlled, and trimmed against other datasets (Figure S1). The processed combinations of sRNA and degradome datasets were analyzed in PAREsnip2 [25] in order to generate cleavage prediction of potato and *P. infestans* transcripts in all possible combinations (Figure S2). Potential Pi-sRNA cleavages in uninfected leaves and St-sRNA cleavages in *P. infestans* mycelia were computed to generate “negative” control information used to refine the other datasets. The output of PAREsnip2 was imported into R for true cleavage identification by the smartPARE R package and filtered to only include true positives. Final datasets were generated by comparing normalized fragment abundance (NFA) difference between infected and control samples (Table S2).

### 2.2. A total of 7.3% of All Cleavage Windows Represented True Cleavages

**Figure 1:**
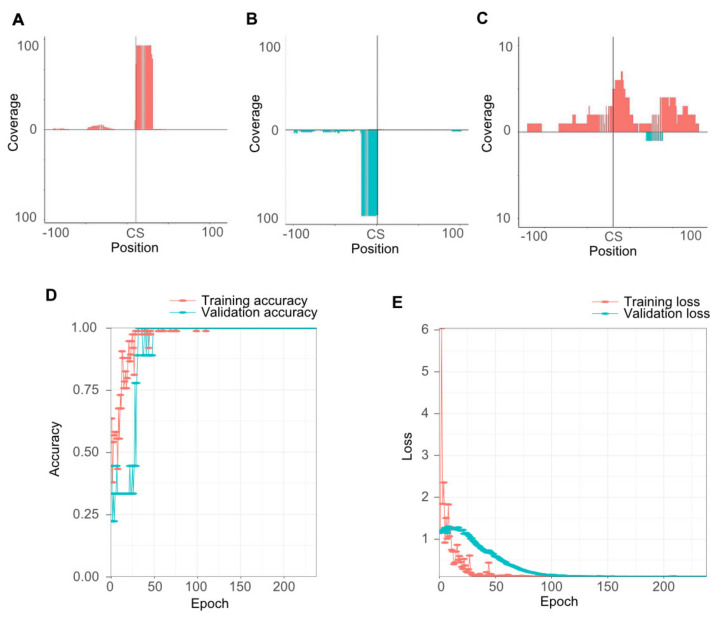
Training of cleavage recognition. (**A**) Cleavage plot displaying a true cleavage at the 5′ Whatson strand. (**B**) Cleavage plot displaying a true cleavage at the 3′ Crick strand. (**C**) Cleavage plot displaying false cleavage at the 5′ strand. Reads on the 5′ strand in red and on the 3′ strand in blue. CS = Proposed cleavage site by PAREsnip2. (**D**) Training accuracy (red) and (validation accuracy) per epoch of the final convolutional neural network (CNN) model. (**E**) Training loss (red) and validation loss (blue) per epoch of the final CNN model.

From the sRNA and degradome datasets, PAREsnip2 predicted in total 32,886 mRNA cleavages. An R function was constructed to view BAM alignments of the predicted target sites in sRNA cleavage plots (Figure 1A–C), generating windows for every cleavage (one for each degradome library). Characteristic cleavage was seen (Figure 1A,B), but more commonly background noise regarded as false information (Figure 1C). To automatize identification of true cleavages among the resulting 65,772 cleavage plot windows, we developed a deep learning convolutional neural network (CNN) using the R interface to Keras [24]. To develop training categories for the model, we visually interpreted and classified different categories of cleavage events as false predictions (305 events), true predictions on the 3′ strand (108 events), and true predictions on the 5′ strand (90 events). A higher number of false predictions were chosen in this step since they contained more variation, resulting in a more robust validation of the model (Figure 1D,E).

**Figure 2:**
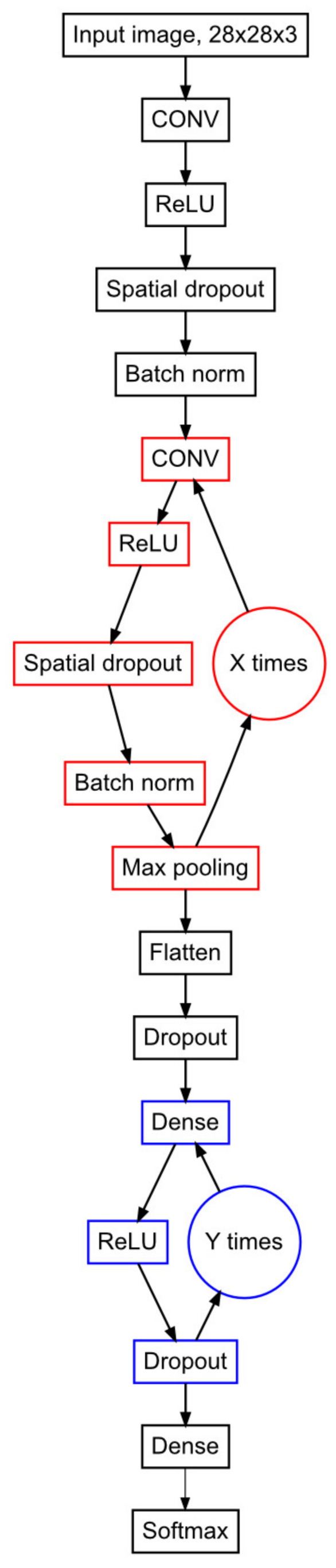
Flowchart of the convolutional neural network (CNN) model. CONV is a 2D convolutional layer with a L1 regularization factor of 1e-4. ReLU is the rectified linear activation function. Spatial dropout is a 2D spatial dropout layer, dropping entire 2D feature maps. Batch norm is a batch normalization layer, normalizing the activation of the previous layer at each batch. Numbers of iterations (X) of the convolutional layers loop (red) is defined by the Bayesian optimization (Table S4, convolutional loop iterations). For every iteration, the number of filters of the loop is doubled. The first and potentially second convolutional layers loop contains a 2D max pooling layer of size 2 × 2. The data are flattened (Flatten) after the convolutional layers loop. Individual element dropout (Dropout) is applied for the rest of the model. The dense layers loop (blue) applies a L2 regularization factor of 1e-4. The number of iterations (Y) of the dense layers loop is defined by the Bayesian optimization (Table S4, dense loop iterations). For every iteration, the number of units is doubled. The model is finalized with a dense layer with as many units as there are different categories of windows (3 in this model) and a Softmax activation step.

Tuning of the hyper-parameters of the deep neural networks is crucial to achieve the most optimized model. In our case, the learning rate is the most important parameter [26]. If the learning rate is too small, it will cause a training algorithm to converge slowly, while if it is too large, the learning rate will infer divergence. A stochastic gradient descent optimizer is defined accordingly: $\theta^{t}=\theta^{t-1}-\epsilon_{t}\frac{\partial L}{\partial \theta }$, where θ = the weights, $\epsilon_{t}$ = the learning rate, and *L* = loss function. A stochastic gradient descent optimizer with cyclical learning rate (CLR) achieves improved classification accuracy [26]. To tune the CLR, it is advisable to run a learning range test. We ran the model for 20 epochs, as this returned a smooth graph and detected the minimum and maximum value for the learning rate (min = 5 × 10^−4^, max = 3 × 10^−2^; Figure S2). To improve the hyper-parameters, we applied Bayesian optimization [27] to the model (Figure 2; Table S3). The probability of random sampling served as rational for the least number of iterations of the Bayesian optimization to use. The probability of random sampling is defined as $p\leq 1-q^{n}$, where q is the desired quantile that should contain the result and n is the number of iterations. Hence, $n\geq \frac{\log\left(1-p\right)}{\log\left(q\right)}$. A desired probability of 0.95 and a quartile of 0.95 resulted in *n* ≥ 59. To increase the probability of a satisfactory model, we used 100 iterations (Figure S3).

In Bayesian optimization, the model is evaluated, within limits specified by the user (Table S4), after each iteration on the basis of a validation score. The evaluation measures of the CNN model, loss, and accuracy correlate negatively. When evaluating the Bayesian optimization, we detected that the best models reached 100% validation accuracy while the validation loss still had not reached zero. Hence, in order to enable the best tuning possible, the inverted loss value was applied as validation score. When this setting was applied, 25 of our 100 CNN models scored 100% accuracy. The model of the 92nd iteration generated the lowest validation loss of 0.10 (Figure 1D,E; Table S4), and was used in the forthcoming analysis. From the 65,772 cleavage windows evaluated by the model, 4776 were identified as displaying true cleavages (4073 in potato and 702 in *P. infestans*). These cleavages were present in 444 potato transcripts and 214 *P. infestans* transcripts (Figure S4).

### 2.3. Evaluation of smartPARE and Validation of miRNA Cleavage Sites

In order to evaluate our CNN model and its output, we chose two strategies. The first was based on the assumption that the PAREsnip2 tool has an estimated true prediction rate of 90% for miRNAs [25]. If smartPARE performs well, the same level of true miRNA cleavages should be identified among the PAREsnip2 cleavage candidates. To test this hypothesis, we retrieved *Arabidopsis* degradome raw data from the hypomorphic *ago1–27* mutant and the wild-type (Col) together with *Arabidopsis* miRNAs from www.pmiren.com (accessed on 25 September 2020) and analyzed them with smartPARE, applying our trained model. The *ago1–27* mutant is proposed to produce sub-optimal cleavages [28] resulting in windows with less distinct cleavage sites, and hence included here to test if smartPARE could also detect these sub-optimal cleavage sites. We found 47 predicted cleavages in the *ago1–27* degradome and 50 in the wild-type degradome. Thus, all PAREsnip2-identified cleavages in *Arabidopsis* were categorized as true by smartPARE. In cleavage windows, an miRNA–mRNA cleavage does not differ to other sRNA cleavages, suggesting that reliable true cleavages are detected when applying smartPARE.

Next, we searched for potato miRNAs in our datasets and their induced mRNA cleavage sites being supported by previous studies. Seven candidates were found (Figure S5; Table S5). Besides miR159 that repress *GAMBY-like* genes encoding MYB domains [29], we detected miR156 activity with a preference for targeting *SQUAMOSA-promoter binding-like* (*SPL*) transcription factors, similar to the known targets of this miRNA family in *Arabidopsis* and rice [30,31]. Furthermore, we detected cleavage events induced by miR160, which targets *auxin response factor* (*ARF*) genes in potato, similar to their function in *Arabidopsis* [32]. Additionally, gene regulation by miR164 of PGSC0003DMT400050262, a *NAC* transcription factor, and miR166 of *HD-ZIPIII* genes were found. miR403 and miR6024 were also found to regulate the potato homologs of *AGO3* and *Rx*, respectively [33,34]. Altogether, the detection of these conserved miRNA-targeting events demonstrates the detection strength of smartPARE and its reliability to analyze sRNA-mediated targeting events using PARE libraries.

### 2.4. Crinkler Effectors Are Involved in sRNA Cascades during Infection

To detect infection-induced changes in sRNA cleavage activity of potato and oomycete transcriptomes, we compared cleavage depth (estimated as NFA) between each pair of infected and control datasets. Below, we present NFA cleavage data since we believe it gives a more correct picture and a better understanding of biological functions of the identified mRNA targets. By this approach, the two categories of resistance genes (73) and transcription factors (74) were the largest by numbers with increased NFA upon infection (Figure S6A–C) when considering St-sRNAs targeting potato mRNAs. Among the Pi-sRNAs targeting potato mRNAs with increased NFA upon infection enzyme and hormone groups were the greatest (13, 4; Figure S6D). Resistance genes and transcription factors were also the top candidates among the sRNA with decreased NFA upon infection (62, 21; Figure S7). When we analyzed the activity of Pi-sRNAs against *P. infestans* reference transcriptome (ASM14294v1.33)*,* a difference in NFA was only detected in the PiAgo1//Pi-sRNA datasets (Figure S8). Here, most targets were ribosomal RNAs followed by enzymes, both within datasets with increased and decreased NFA. Among the genes with increased NFA, an RXLR (PITG_04760)-derived 21 nt sRNA was found to target an N-acetyltransferase (PITG_14006). Additionally, two intergenic iso-sRNAs (length and/or sequence variant sRNA) sequence variants of 20 and 21 nt (supercontig 1.12) were targeting an RNA polymerase (PITG_08173). The shared second largest groups with increased NFA were transporters (PITG_04261—ABC transporter, PITG_08226—amino acid-polyamine-organocation) and effectors (PITG_04760; RXLR and PITG_04768; Crinkler). Interestingly, we identified a 21 nt sRNA targeting PITG_04760 (RXLR transcripts) derived from PITG_0475, potentially triggering biogenesis of secondary siRNAs. Surprisingly, none of the siRNAs involved in this event were detected in our PiAgo1//Pi-sRNA mycelia control, suggesting that this is an infection-triggered cascade. In summary, sRNA cleavage activity is highly dynamic during *P. infestans* infection and targets a diverse array of plant genes with functions related to defense.

### 2.5. The Complex Small RNA Landscape during Potato–P. infestans Interaction

**Figure 3:**
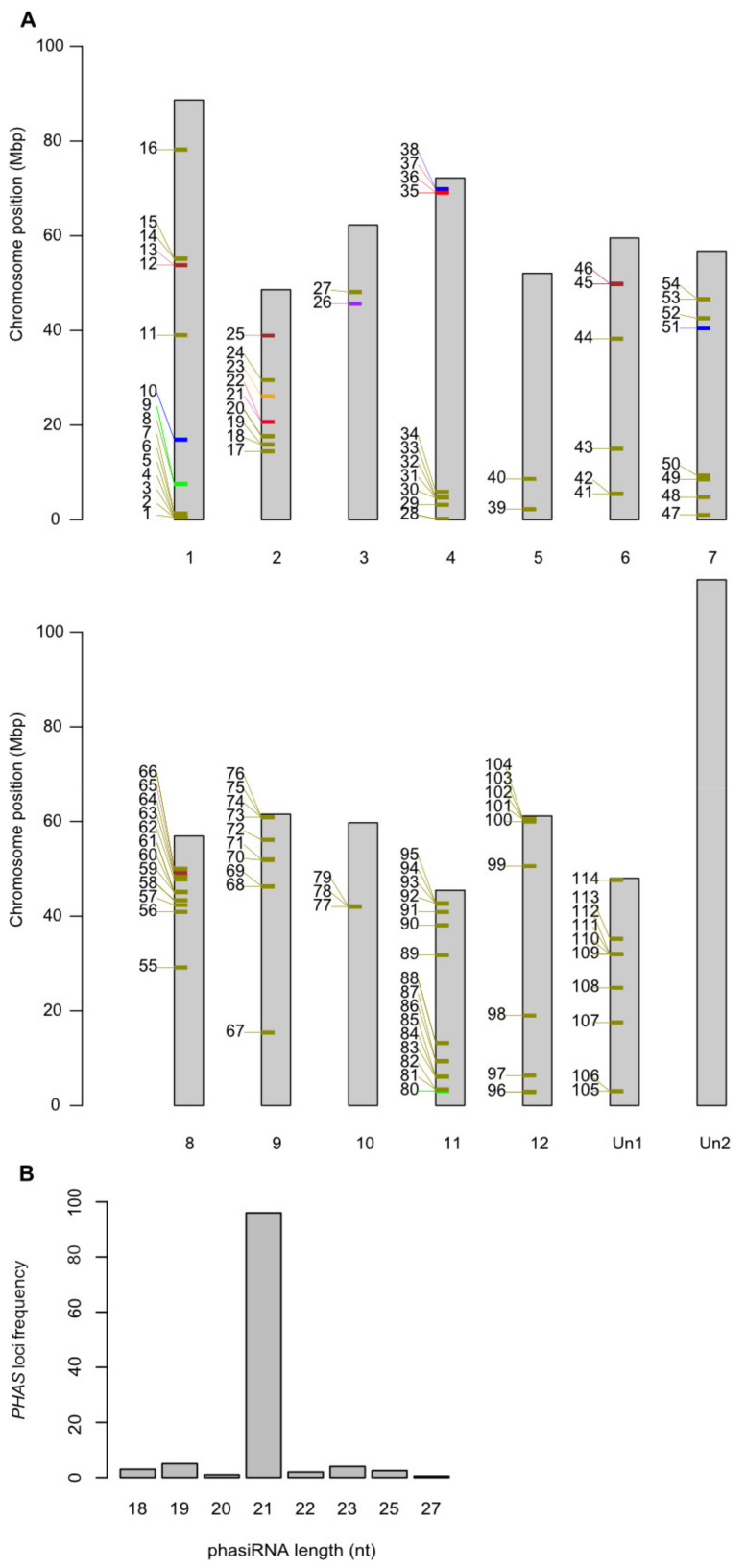
*PHAS* loci and size frequency. (**A**) One hundred and fourteen *PHAS* loci were predicted by Phasetank from all sRNA reads included in the analysis and mapped on the potato chromosomes. Colors illustrate phasiRNA nt length (18 = blue, 19 = red, 20 = orange, 21 = dark gold, 22 = green, 23 = brown, 25 = purple, 27 = black). Un1 and Un2 are the unanchored sequences from the potato genome version 4.03 and 4.04, respectively. (**B**) Frequency of *PHAS* loci in relation to phasiRNA length (nt). One locus produced both 25 and 27 nt sRNA, and hence each nt length was assigned 0.5 for that loci. Details on the different *PHAS* loci are given in Table S8.

Next, we took advantage of our extensive sequencing of sRNAs from potato to identify the changes in different categories of its sRNAs upon infection. When summarizing the total number of targeting sRNAs, we discovered 222 endogenous Pi-sRNAs, 566 endogenous St-sRNAs (out of which 165 were isomiRs from 20 different potato miRNA families), 91 translocating Pi-sRNAs, and 14 translocating St-sRNAs (nine isomiRs from a single miRNA) (Tables S6 and S7). We next took a closer look at phased secondary siRNAs (phasiRNAs) and tasiRNAs. PhasiRNAs are important regulators of stress responses generated from *PHAS* genes after cleavage by miRNAs. To maximize identification of phasiRNAs in the potato genome, we merged and defined all St-sRNAs as a single sRNA dataset. Likewise, all degradome reads formed a degradome set. This approach identified 309 phasiRNAs from 114 *PHAS* loci (39 from non-coding precursors) unevenly distributed on the potato chromosomes (Figure 3A). The length of phasiRNAs spanned between 18 and 27 nt with a peak of 21 nt, as expected from DCL4-homologous activity (Figure 3; Table S8).

**Figure 4:**
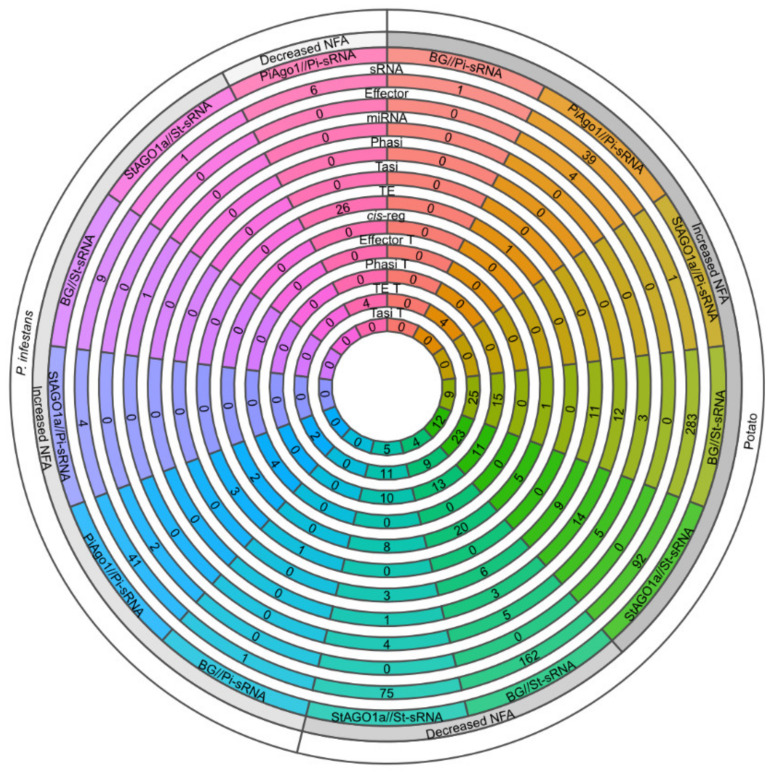
Precursor and target site summary (number of genes excluded) of sRNA targeting in potato and *P. infestans*. White ring: the targeted genome. Gray ring: specifies increased or decreased NFA upon infection. Subsequent rings towards the center corresponds to different materials, RNA classes, and number of sRNAs related to each class. All classes denoted ¨T¨ in the end implies that the class corresponds to the target site. TE (transposons and repeats), *cis*-reg (*cis*-regulatory sRNAs), phasi (phasiRNAs), and tasi (tasiRNAs) are according to [1].

An overview of endogenous and exogenous sRNAs during the potato–*P. infestans* interaction is visualized in Figure 4. Most tasiRNAs (phasiRNAs acting in *trans*, derived from *TAS* loci) were detected in the datasets with increased NFA (11 in BG and 9 in StAGO1a). The St-derived sRNAs from the background sequencing contained most cleaving sRNAs in potato (283 increased NFA, 162 decreased NFA values). In the background dataset with decreased NFA values, the majority of the *cis*-regulatory sRNAs in potato were found. Fifteen of these *cis*-regulatory sRNAs derived from two loci: PGSC0003DMG400022689 and PGSC0003DMG400013938. miR159c-3p (20–22 nt) isomiRs targets a *GAMYB-like* gene (PGSC0003DMG400022689), a miR159 cleavage also reported in *Arabidopsis* [29], and a phasiRNA locus in a gene of unknown function (PGSC0003DMG400013938), generating 20 and 21 nt phasiRNAs (Figure S9A).

**Figure 5:**
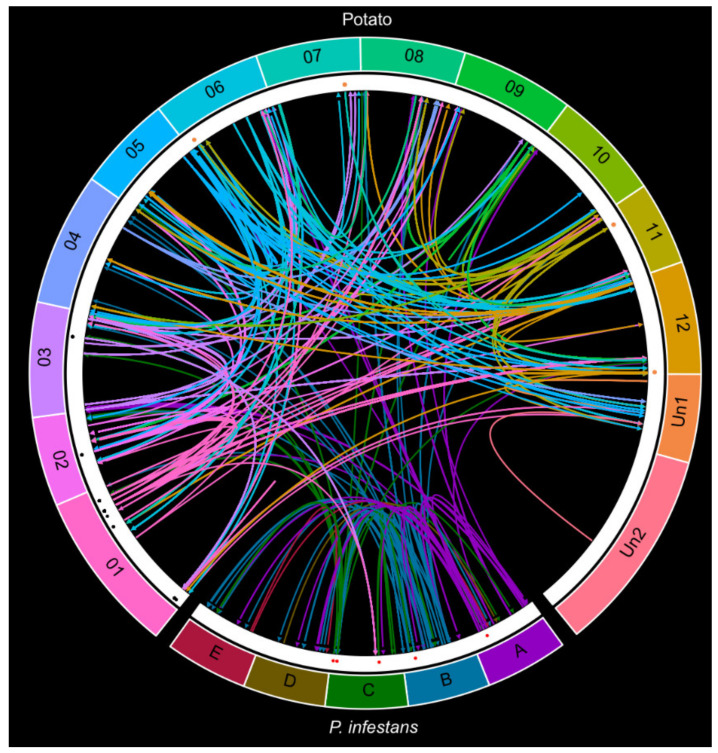
Precursor and target sites of endo- and exogenous sRNAs in NFA-increased datasets. Organized chromosome vice (potato) or as groups of supercontigs (A–E) for *P. infestans*. Un1 and Un2 are the unanchored sequences from potato genome version 4.03 and 4.04, respectively. The arrows represent the connection between each source and target (arrowhead) and are colored according to the precursor chromosome or region. Colored dots in the white margin represent the following category of source sRNA loci: phasiRNAs (orange), TE (red), miRNAs (black).

A Pi-miRNA have been described to target host mRNA during infection [23]. We investigated the presence of such *trans*-kingdom sRNA activity in our new datasets and, indeed, we detected 39 sRNAs present in the PiAgo1//Pi-sRNA dataset that cleaved potato mRNAs. Four of these Pi-sRNAs originated from RXLR effectors, and one from a *Gypsy* transposon. Four intergenic sRNAs (two from supercontig 1.52 and two iso-siRNAs from supercontig 1.2) were targeting LTR and *SINE* transposons in potato. Pi-sRNAs from the PiAgo1//Pi-sRNA dataset with increased NFA contained most endogenous mRNA-cleaving sRNAs, ranging from 19 to 22 nt. This group contained, for example, 19–21 nt iso-sRNAs generated from two RXLR effectors (PITG_04760 and PITG_04764), two self-regulatory iso-sRNAs (20 and 21 nt) from PITG_12253 (with unknown function), and four RXLR effectors being targeted by sRNAs (19 and 21 nt) upon infection. In the group of Pi-sRNA with decreased NFA, four iso-sRNAs (19, 20, 21, and 23 nt) generated from one *Gypsy* sequence cleaved a single DNA-type transposon (EPrPINT00000000324) and a repeat in the group of unknown type 3 (EPrPINT00000005379). Among the St-sRNAs targeting *P. infestans*, only a single mRNA with known protein function, a putative adenylate cyclase (PITG_10287) was found. Interestingly, this particular St-sRNA was derived from an *R* gene (PGSC0003DMG400030207). A summary of all genomic mapped precursor transcripts and targets of various sRNA classes identified in this study is illustrated (Figure 5; Figure S10; Datasets 1 and 2).

### 2.6. A cis-Regulating R Gene Induces a Three-Step phasiRNA Cascade

Our approach identified sRNA-related events taking place in the potato–*P. infestans* interaction that involved single and multiple cascade patterns. Regarding Pi-sRNAs, we detected two supercontigs (1.6 in region B and 1.52 in region C) that produced a high density of Pi-sRNAs (30% jointly, ranging between 19 and 21 nt), all targeting potato transcripts (Figure 5). An example of such cascade event started with two 21 nt sRNAs targeting a *P. infestans* rRNA gene (EPrPINT00000003753) at two positions (423 and 2209) (Figure S9). These targeting events induced the production of 20 nt sRNAs and two iso-sRNAs from *P. infestans* (19 and 21 nt) that targeted a gene of unknown function in potato (PGSC0003DMT400032714) and a gene of unknown function in *P. infestans* (PITG_22016) (Figure S9B). In a similar fashion, another rRNA (EPrPINT00000002574) at supercontig 1.52 in *P. infestans* was targeted by a sRNA from an intergenic site at supercontig 1.21. The same rRNA generated a sRNA that targeted a potato zinc-finger (PGSC0003DMT400026178) mRNA (Figure S9C). These examples demonstrate a complexity of the regulatory cascades during *P. infestans* infection that were unknown previously.

**Figure 6:**
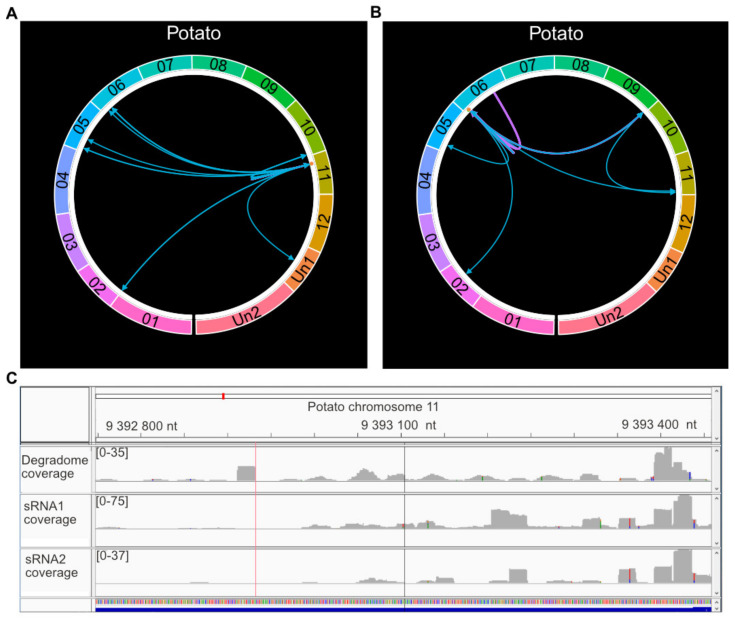
Precursor and target sites of sRNAs in cascade events. (**A**) Two-step event, first involving the *R* gene PGSC0003DMG400030207 followed by the *R* genes PGSC0003DMG400002357, PGSC0003D MG400002426, PGSC0003DMG400009633, PGSC0003DMG400009686, PGSC0003DMG400018429, and two PHD finger transcription factor-coding genes (PGSC0003DMG400018039, PGSC0003 DMG400023718). (**B**) Three-step event involving the following genes: Step 1: The *R* gene PGSC0003 DMG400008697. Step 2: seven *R* genes (PGSC0003DMG400007743, PGSC0003DMG400008697, PGSC0003DMG400013482, PGSC0003DMG400021216, PGSC0003DMG401026432, PGSC0003DMG400026433, PGSC0003DMG400033670). Step 3: a histidine-rich glycoprotein-encoding gene (PGSC0003DMG400040934). The circles are organized chromosome vice (potato). Un1 and Un2 are the unanchored sequences from the potato genome version 4.03 and 4.04, respectively. The arrows represent the connection between each precursor and target site (arrowhead). The orange dot in the white margin represents phasiRNAs (precursor loci). The first step of the cascade (the triggering event) is symbolized with a purple arrow. The arrows representing secondary events are turquoise. (**C**) An Integrative Genomics Viewer (IGV) screenshot of a *PHAS* locus in gene PGSC0003DMG400030207. Top panel represents the degradome coverage (leaves cv. Binje, *P. infestans* wild-type strain 88069). Middle and bottom panels show two sRNA replicates from StAGO1a (St-sRNA from leaves cv. Sarpo Mira, *P. infestans* wild-type strain 88069). Red line represents the target of a *cis*-regulatory sRNA at position 9392932, 3′ strand. Black line represents the target of a *cis*-regulatory sRNA at position 9393105, 3′ strand.

We were also able to detect several cases of complex regulatory cascades in potato. For example, a *PHAS* locus in the potato *R* gene (Avr9/Cf-9, PGSC0003DMG400030207) induces three 21 nt phasiRNAs (Figure 6A). These three phasiRNAs are *cis*-regulatory and are also predicted to regulate other five *R* genes and two PHD finger transcription factor-coding genes (PGSC0003DMG400018039, PGSC0003DMG400023718). Our approach also identified a three-step cascade (Figure 6B,C). Intergenic iso-sRNAs (20–23 nt) targeted the *R* gene PGSC0003DMG400008697 at two positions (579 and 2475). The very same iso-sRNAs are also generated by the *R* gene itself, and hence a potential self-regulatory step could be induced by this *R* gene. The same *R* gene also generates 19–23 nt sRNAs targeting transcripts in seven additional *R* genes. Finally, in the third step, one of the seven *R* genes (PGSC0003DMG400007743) produced a 21 nt phasiRNA that regulated a histidine-rich glycoprotein on chromosome 11.

### 2.7. Resistance Genes Are a Major Target Group in Potato

In previous work, 755 *R* genes were identified in potato [35]. We used these 755 *R* genes as trasncriptome information in PAREsnip2, followed by filtering using smartPARE. We discovered that infection triggered the production of a high number endogenous potato sRNAs, comprising miRNAs, phasiRNAs, tasiRNAs, and TE-derived and effector-derived sRNAs, with the capacity to silence *R* genes (Figure S11; Table S9). We further searched for trans-kingdom ability of Pi-sRNAs to target *R* genes and found that only one *R* gene is targeted by oomycete sRNAs (Figure S11). When investigating NFA distribution, we found it was broadly equal; 37 *R* genes with increased and 38 with decreased NFA values (Tables S9 and S10). The presence of potato reads in the PiAgo1 dataset was much lower compared to the StAGO1a dataset (Table S1). Still, the PiAgo1 dataset contained most sRNAs cleaving potato *R* genes (Figure S12). This observation suggests that the PiAgo1 protein is unable to distinguish endogenous from exogenous sRNAs. Further, one RXLR effector (PITG_01398) generated a Pi-sRNA-targeting mRNA of a bacterial spot disease resistance protein 4 homolog in the potato genome (PGSC0003DMG400033334) (Figure S13; Table S9), suggesting dual effector functions in abating defense responses. In summary, our degradome analysis identified extensive targeting of defense genes mediated by endogenous (potato) and exogenous (*P. infestans*) sRNAs. Contrasting types of sRNA display divergent but substantial importance in complex cascades and networks.

Resistance genes and other genes known to be involved in plant stress responses are regulated by phasiRNAs triggered by miRNAs cleavage [36,37]. An exploration of these events in our datasets (using PhaseTank) identified 13 triggering miRNAs that mediated cleavage of 17 of the 114 *PHAS* loci. Eight of these miRNAs were 22 nt, indicating general miRNA-mediated cleavage in line with the “one-hit” model [38]. The remaining miRNAs were 20 nt (1), 21 nt (1), and 24 nt (3). Only 1 of these 13 miRNAs were detected among our sRNA reads in any of the datasets (miR6023). This indicates that several AGOs are possibly involved in the miRNA-mediated cleavage of *PHAS* and *TAS* loci in our study-system. Under the assumption that all miRNAs are discovered, a possible explanation for the remaining 85% of *PHAS* loci lacking trigger-miRNA is that other sRNAs are involved in the biogenesis of phasiRNAs. We identified 22 *PHAS* loci transcripts targeted by other types of sRNA, 17 intergenic sRNA, 8 from protein-coding genes (wherein 3 are *R* genes), and 1 Pi-RNA (from PITG_08165).

## 3. Discussion

The high original false-positive rate of 93% (60,997 of 65,772 cleavage windows) in our potato and *P. infestans* datasets forced us to develop a new analytic strategy, an R package to be applied after the degradome analysis tool. SmartPARE was based on a deep learning CNN algorithm in the R interface to the Python package Keras in order to distinguish between true and false cleavages. The CNN model was constructed to contain two loops, one for convolutional layers and the other for dense layers. The loops were designed to expand the number of layers by a factor of two for each iteration. The number of convolutional layers and dense layers are often manually optimized, costing a significant amount of working time as the full model needs to be finalized in order to evaluate the performance of the included functions. When integrating the mentioned loops with the aid of Bayesian optimization [39], estimation of optimal training parameters (hyperparameters) could effectively and efficiently be performed. With further optimization using cyclical learning rate, the optimal learning rate could automatically be determined. Cyclical learning rate and Bayesian optimization are both complex but superior methods and enable training of a model with outstanding cross-validation accuracy (100%). When using this new model to evaluate our data, we found 214 cleavages in *P. infestans* and 444 in potato. Among the cleavages, several 21 nt sRNA cascades were seen, for example, those involving phasiRNAs. Our data also suggest that RXLR and Crinkler-derived sRNAs regulate endogenous genes in potato, opening up for a dual effector functionality both as effector proteins and as sRNA source. The possibility to apply our CNN model, or a self-created CNN model from our script, as a filter step after any degradome analytic tool generates a significantly reduced positive rate. The value in discarding approximately 90% of the PAREsnip2 output data, consisting of false positives, will make degradome analyses of sRNAs more attractive not least when handling large and complex genomes and interests beyond identifying miRNAs. One limitation with degradome analyses beside quality of input materials, library construction, and numbers is the depth (NFA) of the sequencing, a step which covers the whole genome, including all targets and possible degradation products that are poly(A)-marked [40]. Rare targets and low number of fragments (depending on the depth of the sequencing) will not be distinguished from the background noise. In our CNN model, cleavages with a depth less than five reads are discarded. This limit can be adjusted but leads inevitably to higher number of false target candidates. One example of few degradome reads is the predicted target of the single 21 nt miRNA of *P. infestans* targeting a potato mRNA coding for a tonoplast localized protein confirmed by 5′RACE [23]. Likewise, the miR482 family is known to target several *R* genes in *Solanaceae*, including *RB*, *R2*, and *R3a* acting against *P. infestans* [33], but too few degradome reads were found in our datasets to support such targets. When evaluating the sensitivity of window selection in our model, we found that 7 out of 194 cleavage windows (3.6%) were only identified in one of two of the individual libraries. This may indicate that the settings could be too sensitive when distinguishing background from “real” events. To add cleavage plots with increased variations from other species and tissues to the training data might improve the recognition further.

In conclusion, smartPARE was developed to discriminate false from true sRNA-incited cleavage sites, particularly suitable for large genomes. In our case study of the potato–*P. infestans* interaction, smartPARE revealed a complex intertwined regulatory role of sRNA-associated intra- and inter-organismal events.

## 4. Materials and Methods

### 4.1. Plant Materials, Infection, and Growth Conditions

*StAGO1a* (PGSC0003DMG400026963) was amplified using forward 5′CACCATGGTGCGGAAGAGGAGAAC-3′ and reverse 5′CTAACAATAGAACATC ACCCTTTTGAC-3′ primers on potato cDNA. A LR reaction was performed prior to gateway cloning using Gateway LR Clonase Enzyme Mix kit (Thermo Fisher Scientific) and the destination vector pGWB406 [41]. Sanger sequencing was performed to confirm the sequences in the plasmid. Potato transformation was performed as described previously [22]. Leaves from transgenic plants inoculated with *P. infestans* strain 88,069 or water were collected 5 days post-inoculation. Three replicates were prepared for each sample consisting of 2 g of pooled leaf material. sRNA libraries (background) were made through the following procedure. Two replicates of potato leaves (cv. Sarpo Mira) inoculated with 88,069 (Wt) were collected 5 days post-inoculation (dpi). Leaves inoculated with H_2_O were used as control. Each sample consisted of 3 leaves. Total RNA was extracted for small RNA sequencing using the Plant/Fungi total RNA purification kit (Norgen; Ontario, Canada). Four sRNA libraries were generated using Illumina small RNA sample preparation kit. Libraries were prepared by SciLife sequencing platform (Stockholm, Sweden) under their in-house conditions and sequenced using Illumina HiSeq 2500. Data analysis was performed as described in Figure S1. Plant growth conditions, pathogen storage, cultivation conditions, and inoculation procedures were the same as outlined previously [13,22].

### 4.2. Library Preparations

Co-immunoprecipitation and sRNA sequencing were performed as described previously [23]. For degradome analysis, 2 replicates of potato cv. Bintje leaves inoculated with either H_2_O or any of the 2 *P. infestans* strains 88,069 or *PiAgo1-GFP* were collected 5 days post-inoculation. For each replicate, 2 g of leaf material was pooled. Mycelia of the 2 *P. infestans* strains were grown in pea broth for 7 days, before collecting 2 mycelia replicates of 200 mg from each strain. Degradome library preparation was performed as outlined previously [42]. All libraries were sequenced at SciLifeLab (Uppsala, Sweden) using HiSeq2500 rapid SR50 technologies.

### 4.3. Degradome Data Analysis

The adaptor trimming and filtering of degradome data was performed with Cutadapt v. 2.4 [43], retaining 3′ anchored adaptor trimmed reads, allowing for a maximum error of 10%. Quality cutoff was set to 20. PAREsnip2 was used to predict small RNA targets from the degradome and sRNA data from the “all”, StAGO1a, and PiAgo1 datasets [25], with default stringent parameters and Fahlgren and Carrington targeting rules [44] in 3 transcriptomes: the potato reference genes [45], the *P. infestans* genes [12], and the potato resistance genes previously identified [35]. The PAREsnip2 output was analyzed in R v. 4.0.2 [46]. Transcript cleavage positions were transposed to genome positions using GFF files from each genome.

### 4.4. Additional Datasets

Additional datasets included in our analysis are sRNAs from *P. infestans* 88069 mycelia [17], updated information on *P. infestans* effectors [47], PiAgo1 sRNA datasets [23], predicted tasiRNA loci [48], and potato resistance genes [35].

### 4.5. Design of smartPARE

By implementing Rsamtools, sRNA read information surrounding the cleavage positions was extracted from BAM alignment files generated with Bowtie2 v. 2.3.4 [49]. The alignment positions 1 nt upstream and 21 nt downstream individual cleavage position was interpolated on the *x*-axis of ggplot2, creating cleavage plots displaying read coverage on the *y*-axis (Figure 1A–C). As a cutoff, the minimum depth of cleavage reads on the *y*-axis was set to 5 reads in order to avoid false positives from background noise. Convolutional neural network models were implemented using R interface to Keras v.2.3.0 [50] and TensorFlow v.2.2.0 [51] running through Python v.3.6.10 [52] with NumPy v.1.18.4. For data optimization, the CLR R function was adopted from the code at http://thecooldata.com/2019/01/learning-rate-finder-with-cifar10-keras-r/ (accessed on 29 June 2020) and integrated in the CNN model illustrated in Figure 2. Bayesian optimization R package rBayesianOptimization (v.1.1.0) was further implemented to tune the other hyperparameters in the CNN model. The smartPARE R package was compiled using roxygen2 (v.7.1.1). R packages used are listed in the Methods section of the Supplementary Materials section.

### 4.6. Prediction of phasiRNAs

All St-sRNAs and degradome reads were analyzed by PhaseTank v1.0 [53] in order to generate a potato phasiRNA dataset. For clarification, a sRNA was defined as *cis*-regulatory if targeting its own precursor transcript.

## Data Availability

Raw and processed sequencing data generated in this study were submitted to the NCBI Gene Expression Omnibus (GEO; https://www.ncbi.nlm.nih.gov/geo/) under accession number GSE163382. The smartPARE R package is available online (https://github.com/KristianHoden/smartPARE/), doi:10.5281/zenodo.4495749.

## Supplementary Materials

The following are available online at https://www.mdpi.com/article/10.3390/ijms22084267/s1, Figure S1. Data processing. sRNA pipeline. Figure S2. Workflow of cleavage prediction. Figure S3. Learning rate plotted against accuracy after 20 epochs with rollMean (R package zoo). Figure S4. True target transcripts after conventional neural network (CNN) filtration. Figure S5. Examples of windows surrounding the 7 miRNA–mRNA cleavages confirmed by data from earlier studies. Figure S6. Target genes in potato with increased NFA upon infection organized according to annotated gene functional categories. Figure S7. Target genes in potato with decreased NFA upon infection organized according to annotated gene functional categories. Figure S8. Target genes in *P. infestans*. Figure S9. Precursor and target sites of sRNAs in cascade events. Figure S10. Precursor and target sites of sRNAs in NFA-decreased datasets. Figure S11. Number of true target genes after CNN filtration in the resistance gene version of the PiAgo1//St-sRNA, StAGO1a//St-sRNA, and PiAgo1//Pi-sRNA datasets. Figure S12 Precursor and target site summary (number of genes excluded) of the *R* gene dataset. Figure S13. Precursor and target sites of endo- and exogenous sRNAs in the resistance gene datasets. Table S1. Number of reads after quality filtration in all 15 datasets included in the analysis. Table S2. Comparison datasets (CDs) were created by comparison between infection datasets and non-infection datasets. Table S3. Summary of final model visualizing the shape and number of parameters (param #) for each layer of the model. Table S4. Tunable parameters in the Bayesian optimization. Table S5. miRNA–mRNA cleavage data from earlier studies detected in the present analysis. Table S6. Potato miRNA families. Table S7. Precursor summary of sRNA targeting in potato and *P. infestans*. Table S8. Number of *PHAS* loci per nucleotide length of each individual locus. Table S9. Target genes with increased NFA in the *R* gene dataset. Table S10. Target genes with decreased NFA in the *R* gene dataset. Dataset 1. Precursor and target site information of endo- and exogenous sRNAs in the NFA-increased datasets. Dataset 2. Precursor and target site information of endo- and exogenous sRNAs in the NFA-decreased datasets.
