# Supplementary material for: smartPARE: An R Package for Efficient Identification of True mRNA Cleavage Sites
Source: Int J Mol Sci. 2021 Apr 20;22(8):4267. doi: 10.3390/ijms22084267 (PMC8073297; doi:10.3390/ijms22084267)
Supplement: Supplementary file 1 [file ijms-22-04267-s001.zip › Suppl/Suppl Figs and Tables IJMS-final.pdf]

Supplemental Information

smartPARE: an R package for efficient identification of true mRNA cleavage sites

Kristian Persson Hodén, Xinyi Hu, German Martinez, Christina Dixelius

The Swedish University of Agricultural Sciences, Department of Plant Biology, Uppsala BioCenter, Linnean Center for Plant Biology, P.O. Box 7080, S-75007 Uppsala, Sweden

Contents

Supporting Methods ………………………………………………………………… 3

Supporting Figures …………………….……………………………………………… 4
Fig. S1 Data pre-processing ………………………………………………………… 4
Fig. S2 Cleavage prediction workflow ……………………………………………… 5
Fig. S3 Learning rate plotted against accuracy after 20 epochs with rollMean ……….. 6
Fig. S4 True target transcripts after the conventional neural network (CNN) filtration ... 8
Fig. S5 Examples of windows surrounding the 7 miRNA-mRNA cleavages confirmed by data from earlier studies ………………………………………………………………… 9
Fig. S6 Target genes in potato with increased NFA upon infection organized according to annotated gene functional categories………………………………………………… 10
Fig. S7 Target genes in potato with decreased NFA upon infection organized according to annotated gene functional categories………………………………………………… 11
Fig. S8 Target genes in *P. infestans* (A) Genes with increased NFA upon infection organized according to annotated gene functional categories. ………………………….. 12
Fig. S9 Precursor and target sites of sRNAs in cascade events. ……………………… 13
Fig. S10 Source and targets of endo- and exogenous sRNAs in NFA decreased datasets. ……………………………………………………………………… 14
Fig. S11 Number of true target genes after CNN filtration in the resistance gene dataset 15
Fig. S12 Precursor and target site summary (number of genes excluded) of the *R* gene dataset ……………………………………………………………………………… 16
Fig. S13 Precursor and target sites of endo- and exogenous sRNAs in the *R* gene datasets ……………………………………………………………………………... 17

Supporting Tables ……………………………………………………………………… 18
Tab. S1 Number of reads after quality filtration in all datasets included in the analysis... 18
Tab. S2. Comparison datasets……………………………………………………………… 19
Tab. S3 Summary of final model visualizing shape and number of parameters (param #) for each layer of the model.……………………………………………………………… 20
Tab. S4 Tunable parameters in the Bayesian optimization……………………………… 21
Tab. S5 miRNA-mRNA cleavage data from earlier studies detected in the present analysis ……………………………………………………………………………….
Tab. S6 Potato miRNA families ………………………………………………………. 22
Tab. S7 Precursor summary of sRNA targeting in potato and *P. infestans* …………… 23
Tab. S8 Number of *PHAS* loci per nucleotide length of each individual locus………………………………………………………………………………… 24
Tab. S9 Target genes with increased NFA in the *R* gene dataset……………………….. 25
Tab. S10 Target genes with decreased NFA in the *R* gene dataset………………………. 27

Dataset information …………………………………………………………………. 29

References……………………………………………………………………………. 30

Methods

R packages used in this study

BiocGenerics (v.0.34.0, https://bioconductor.org/packages/release/bioc/html/BiocGenerics.html)

circlize (v.0.4.8, https://cran.r-project.org/web/packages/circlize/index.html)

data.table (v.1.13.0, https://cran.r-project.org/web/packages/data.table/index.html)

dplyr (v.0.8.3, https://cran.r-project.org/web/packages/dplyr/vignettes/dplyr.html)

EBImage (v.4.29.2, https://bioconductor.org/packages/release/bioc/html/EBImage.html)

fftwtools (v.0.9-8, https://cran.r-project.org/web/packages/fftwtools/index.html)

generics (v.0.0.2, https://cran.r-project.org/web/packages/generics/index.html)

GenomeInfoDb (v.1.20.0, https://bioconductor.org/packages/release/bioc/html/GenomeInfoDb.html)

GenomicAlignments (1.20.1, https://bioconductor.org/packages/release/bioc/html/GenomicAlignments.html)

GenomicRanges (v.1.36.1, https://bioconductor.org/packages/release/bioc/html/GenomicRanges.html)

ggplot2 (v.3.3.2, https://cran.r-project.org/web/packages/ggplot2/index.html)

gridExtra (v.2.3, https://cran.r-project.org/web/packages/gridExtra/index.html)

igraph (v.1.2.4.1, https://cran.r-project.org/web/packages/igraph/index.html)

IRanges (v.2.22.2, https://bioconductor.org/packages/release/bioc/html/IRanges.html)

keras (v.2.3.0.0, https://cran.r-project.org/web/packages/keras/index.html)

kerasR (v.0.6.1, https://cran.r-project.org/web/packages/kerasR/index.html)

magrittr (v.1.5, https://cran.r-project.org/web/packages/magrittr/index.html)

pander (v.0.6.3, https://cran.r-project.org/web/packages/pander/index.html)

R6 (v.2.4.1, https://cran.r-project.org/web/packages/R6/index.html)

reticulate (v.1.16, https://cran.r-project.org/web/packages/reticulate/index.html)

rmarkdown (v.2.7, https://cran.r-project.org/web/packages/rmarkdown/index.html)

Rsamtools (v.2.0.3, http://bioconductor.org/packages/release/bioc/html/Rsamtools.html)

reshape2 (v.1.4.4, https://cran.r-project.org/web/packages/reshape2/index.html)

roxygen2 (v.7.1.1, https://cran.r-project.org/web/packages/roxygen2/index.html)

stringr (v.1.4.0, https://cran.r-project.org/web/packages/stringr/index.html)

tensorflow (v.2.2.0.0 https://cran.r-project.org/web/packages/tensorflow/index.html)

zoo (v.1.8-8, https://cran.r-project.org/web/packages/zoo/index.html)

Supplementary Figures

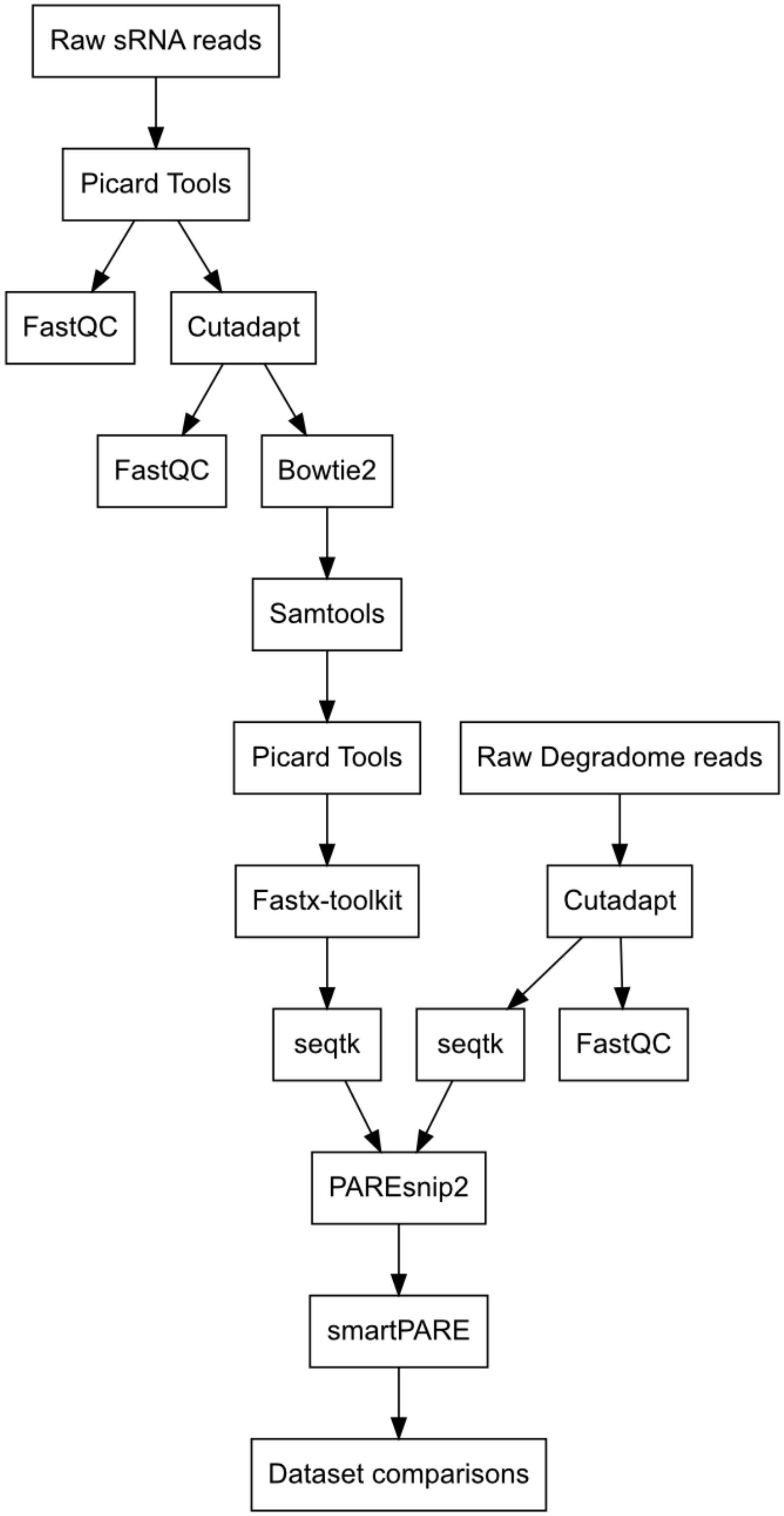

Figure S1 Data processing. sRNA pipeline (left): The function SamToFastq, included in the Picard tool package, was used to convert the original BAM format to FASTQ format (for the raw data which was not already in FASTQ format), on which a quality control was performed, using FastQC. Cutadapt was used for quality trimming with a cutoff of 20 using 18 nt and 38 nt as minimum and maximum read lengths. Bowtie2 in combination with SAMtools view were used for tRNA and rRNA trimming, allowing 0 mismatches per seed against each filtered dataset. The same tools were used to separate potato and *P. infestans* sRNA by first mapping the sRNA to the individual genomes [1,2]. Next, the St-sRNA pool was trimmed against the *P. infestans* genome and the Pi-sRNA pool was trimmed against the potato genome to exclude potential false positives. SamToFastq (Picard Tools) converted the files to FASTQ format. Seqtk was applied to convert the FASTQ files to FASTA. Degadome pipeline (right): Raw RNA reads from degradome libraries were adaptor- and quality trimmed with Cutadapt. The resulting files were converted to FASTA files and together with the St- and Pi-sRNA FASTA files, target sites were deduced applying PAREsnip2. The predicted cleavage sites were analyzed in smartPARE to filter away false predictions. Finally, comparisons between datasets of infected and control materials were performed to generate increased or decreased normalized fragment abundance (NFA) datasets

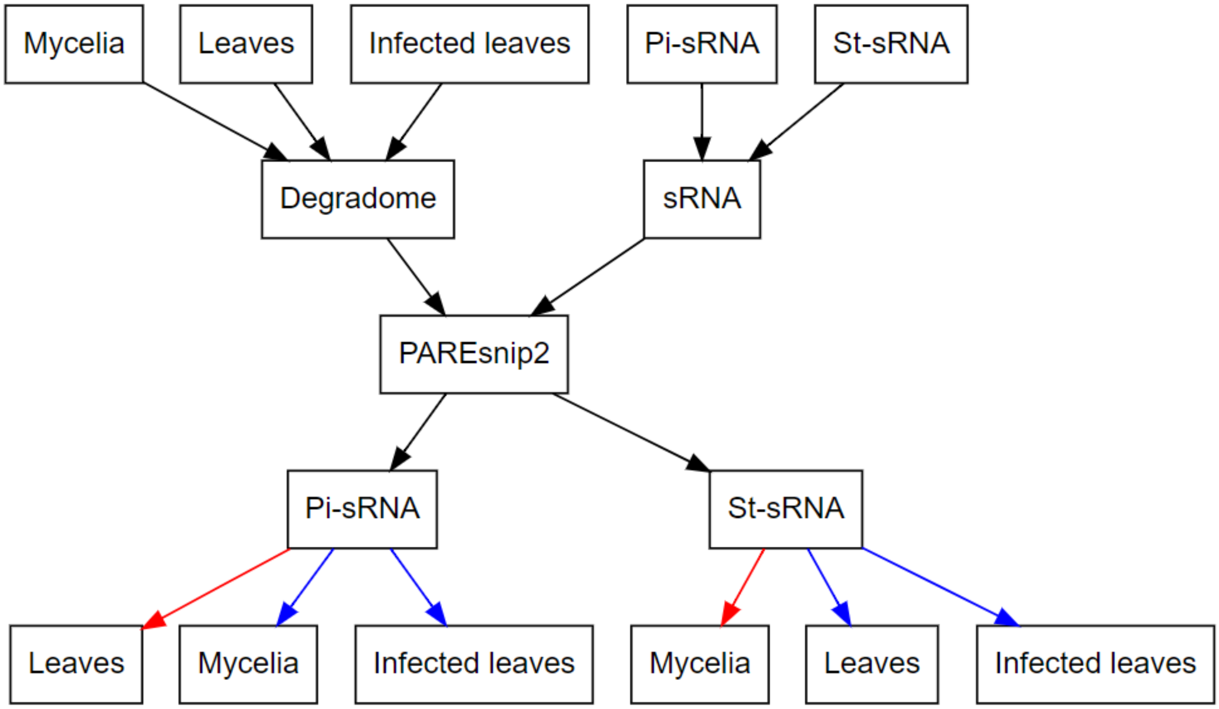

Figure S2 Workflow of cleavage prediction. Different categories of degradome reads (sources: mycelia, leaves and infected leaves) were combined with the different categories of sRNA reads (Pi-sRNA and St-sRNA) in PAREsnip2. Hence, the output datasets of PAREsnip2 consisted of possible (blue arrows) and technical created fallacious (red arrows) combinations. The latter combinations served as negative controls to be filtered against (Supplementary Table S2).

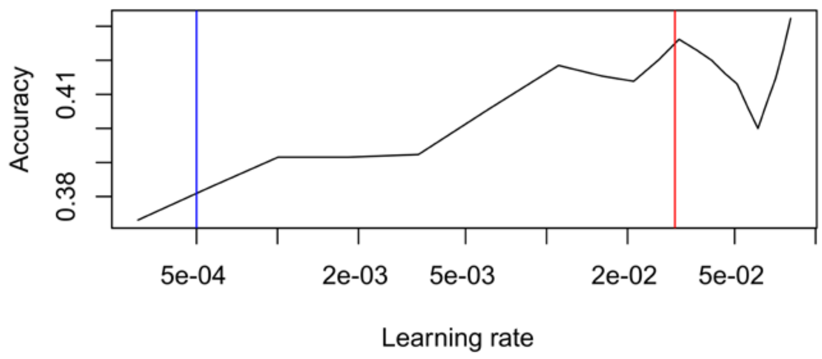

Figure S3 Learning rate plotted against accuracy after 20 epochs with rollMean (R package zoo). The value towards the minimum of the slope (blue line) was set as lower limit ($5x10^{-4}$) in the cyclical learning rate algorithm and the value at the maximum of the slope (red line, $3x10^{-2}$) as the highest.

A

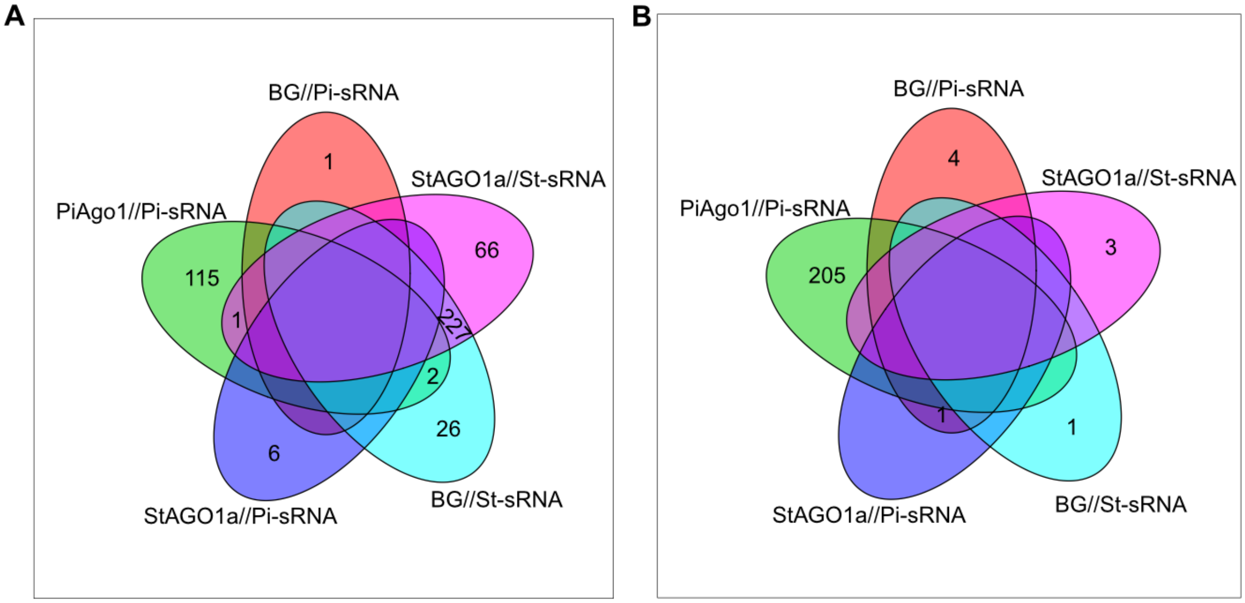

Figure S4 True target transcripts after conventional neural network (CNN) filtration. (**A**) Targeted transcripts in potato by sRNA from the different datasets. (**B**) Targeted transcripts in *P. infestans* by sRNA from the different datasets. Shared cumulative genes are indicated if identified.

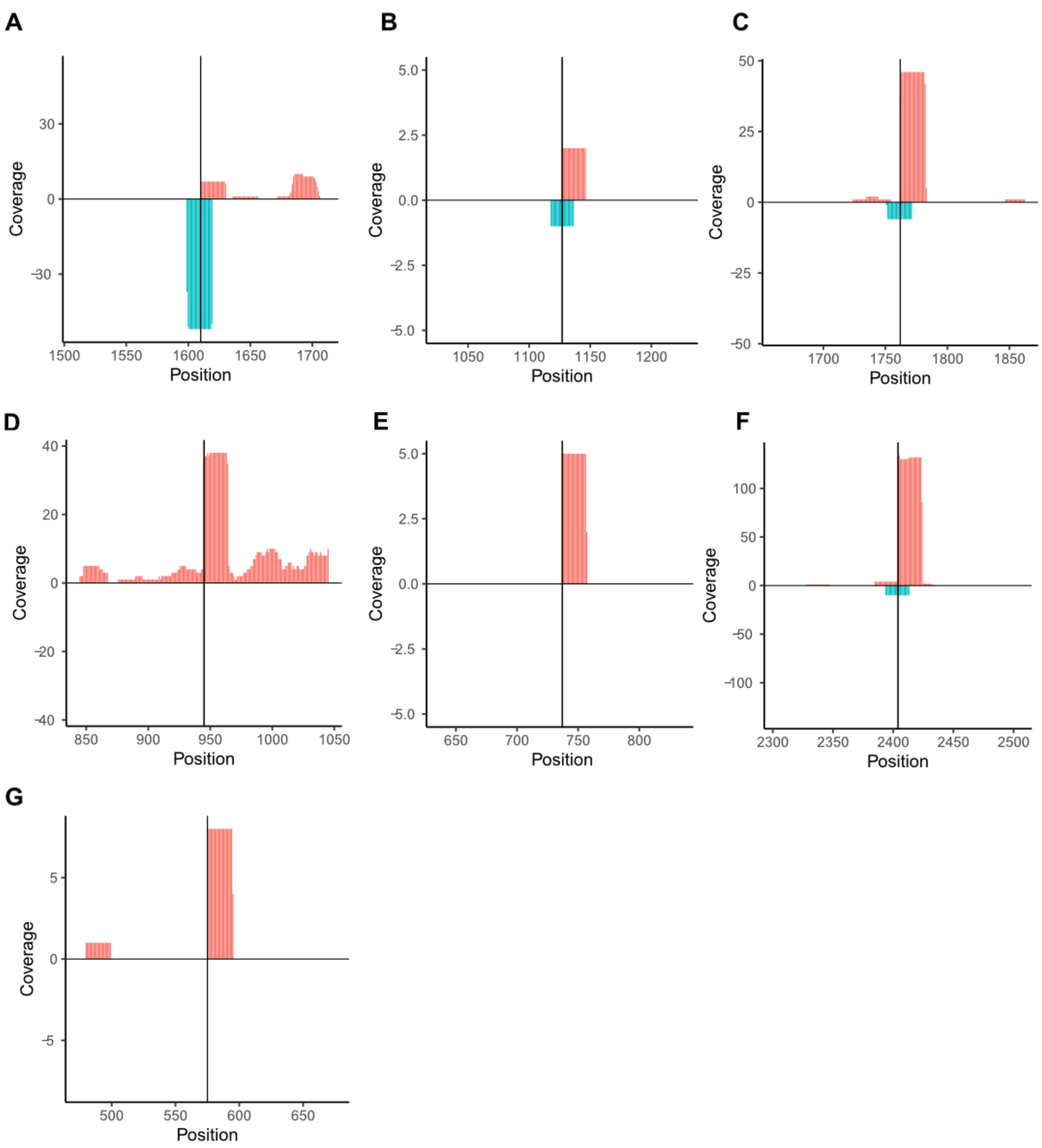

Figure S5 Examples of windows surrounding the 7 miRNA-mRNA cleavages confirmed by data from earlier studies (Table S5). **(A)** miR156d-5p cleaving *SPL* transcript (PGSC0003DMT400076399) at position 1610. (**B**) miR159c-3p cleaving *GAMYB-like* transcript (PGSC0003DMT400058426) at position 1127. **(C**) miR160a-5p cleaving *ARF* transcript (PGSC0003DMT400020874) at position 1762. (**D**) miR164e-5p cleaving *StNAC262* transcript (PGSC0003DMT400050262) at position 945. (**E**) miR166b cleaving *PHAVOLUTA-like HD-ZIPIII* transcript (PGSC0003DMT400030829) position 737. (**F**) miR403b cleaving *Argonaute* transcript (PGSC0003DMT400054667) at position 2404. (**G**) miR6024-3p cleaving *Rx* protein transcript (PGSC0003DMT400005011) at position 575. Reads on the 5' strand in red and on the 3' strand in blue.

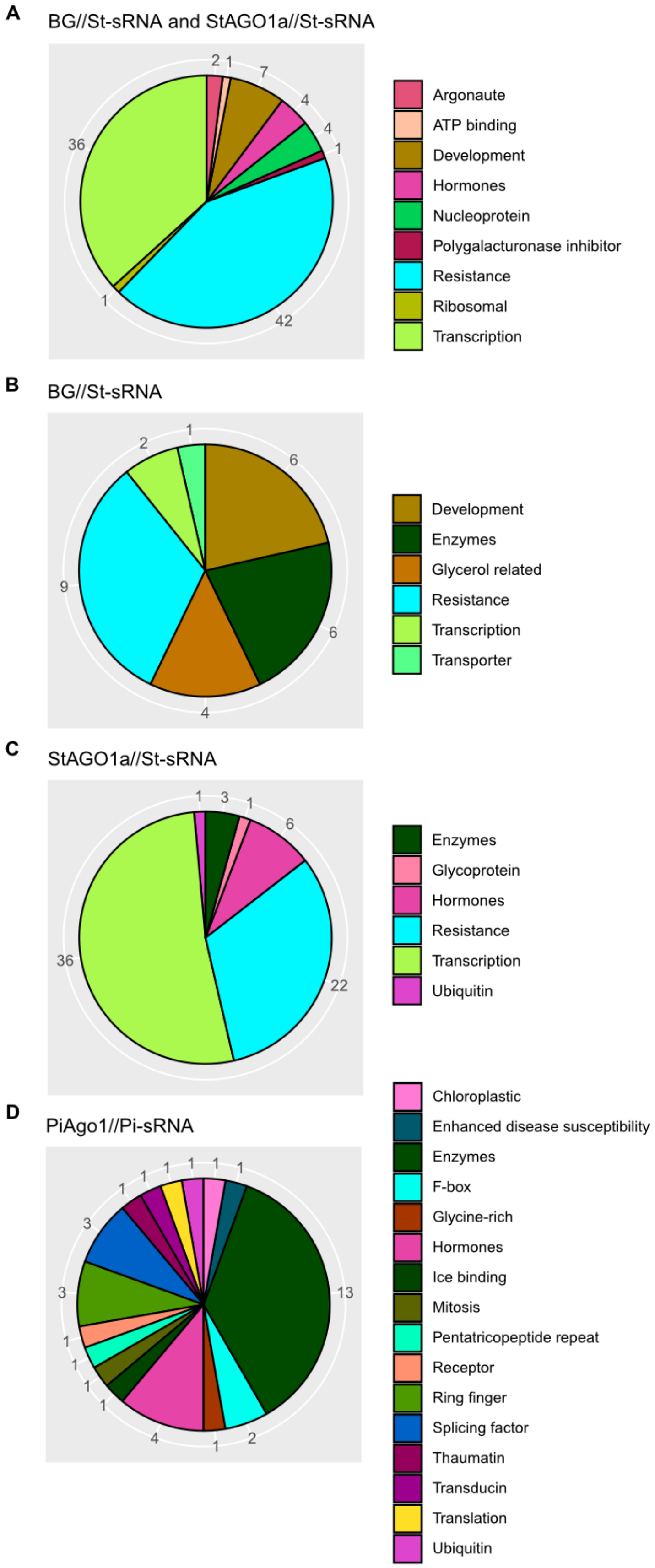

Figure S6 Target genes in potato with increased NFA upon infection organized according to annotated gene functional categories. (**A**) St-sRNA shared target genes between background (BG) and StAGO1a datasets. Four genes of unknown function excluded. (**B**) St-sRNA target genes in the BG dataset. Six genes of unknown function excluded. (**C**) St-sRNA target genes in the StAGO1a dataset. Two genes of unknown function excluded. (**D**) Pi-sRNA target genes in the PiAgo1 dataset, 11 genes of unknown function excluded. Single target functional categories were excluded, they were: BG//Pi-sRNA “zinc finger” (1 target); StAGO1a//Pi-sRNA “Gene of unknown function” (4 targets); PiAgo1 and BG//St-sRNA “hormones” (2 targets).

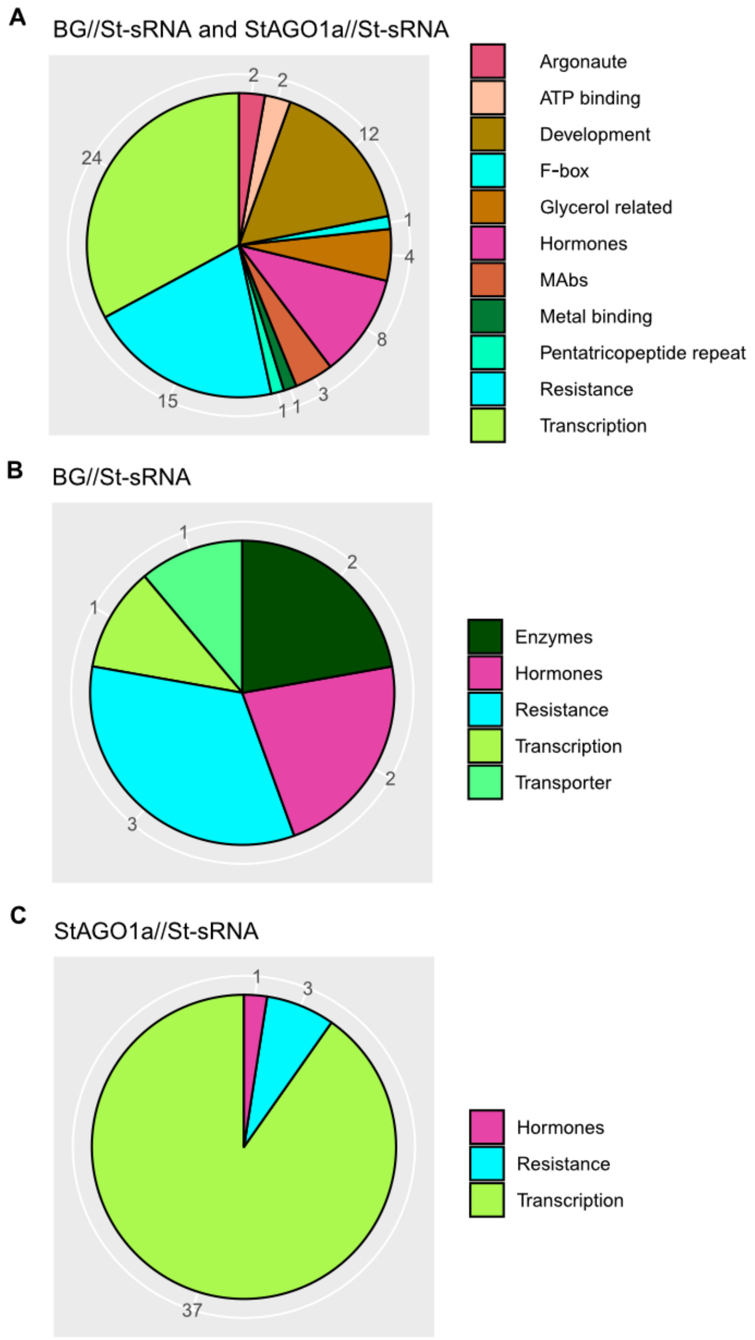

Figure S7 Target genes in potato with decreased NFA upon infection organized according to annotated gene functional categories. (**A**) St-sRNA shared target genes between the BG and the StAGO1a dataset. Ten genes of unknown function excluded. (**B**) St-sRNA target genes in the BG dataset. Four genes of unknown function excluded. (**C**) St-sRNA target genes in the StAGO1a dataset. Single target functional categories and genes of unknown function were excluded, they were: StAGO1a//Pi-sRNA ¨DNA mismatch repair¨ (1 target) and ¨gene of unknown function¨ (1 target).

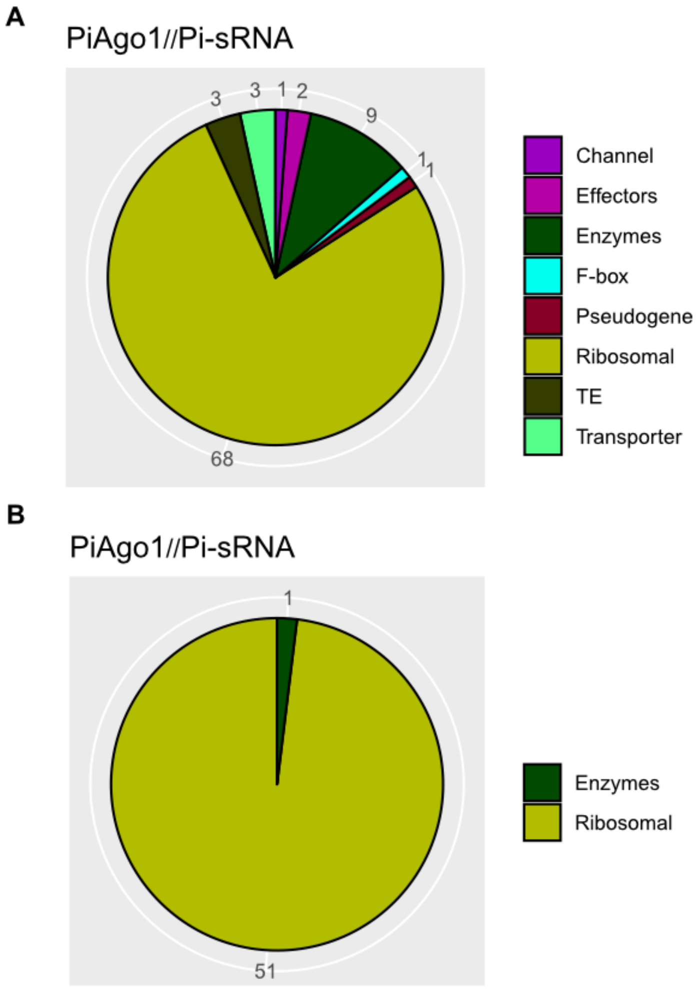

Figure S8 Target genes in *P. infestans.* (**A**) Genes with increased NFA upon infection organized according to annotated gene functional categories. Ten genes of unknown function were excluded. (**B**) Genes with decreased NFA upon infection. Single target functional categories and genes of unknown function were excluded, they were: all//St-sRNA and StAGO1a//St-sRNA "gene of unknown function" (1 target); all//Pi-sRNA and PiAgo1//Pi-sRNA and StAGO1a//P*i*-sRNA "gene of unknown function" (1 target).

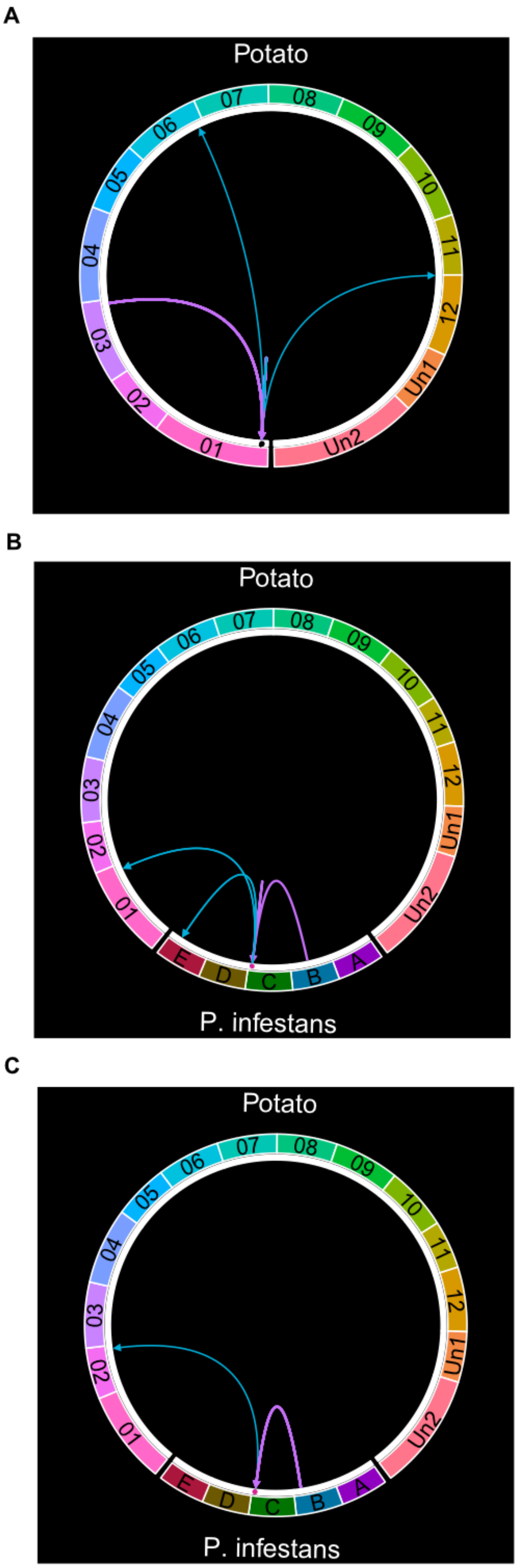

Figure S9 Precursor and target sites of sRNAs in cascade events. (**A**) Intergenic sRNA from chromosome 3 targeting *GAMYB-like1* (PGSC0003DMT 400058425). Self-regulatory miR159c-3p targeting the same position as the intergenic sRNA. A miRNA directed cleavage is also induced on PGSC0003DMT400015155 (*GAMYB-like2*) and PGSC0003DMT400039783 (unknown). (**B**) sRNA from supercontig 1.16 (region B) targeted an rRNA (EPrPINT00000003753) at the supercontig 1.52, between two intergenic loci generating sRNAs. From the intergenic site downstream, a sRNA was found to target a gene of unknown function in potato (PGSC0003DMT400032714). The site upstream of the rRNA generated two iso-sRNAs that targeted a gene of unknown function in *P. infestans* (PITG_22016). (**C**) Trigger-sRNA derived from intergenic site at supercontig 1.21 targeting an rRNA (EPrPINT00000002574) at the supercontig 1.52 derived a sRNA targeting a Zink-finger protein encoding gene (PGSC0003DMT400026178) in potato. The circles are organized chromosome vice (potato) or as groups of supercontigs (A-E) for *P. infestans*. Un1 and Un2 are the unanchored sequences from potato genome version 4.03 and 4.04, respectively. The arrows represent the connection between each precursor and target site (arrowhead). Colored dots in the white margin represent the following category of precursor sRNA loci: rRNAs (dark pink), miRNAs (black). The first step of the cascade (the triggering event) is symbolized with a purple arrow. The arrows representing secondary events are turquoise.

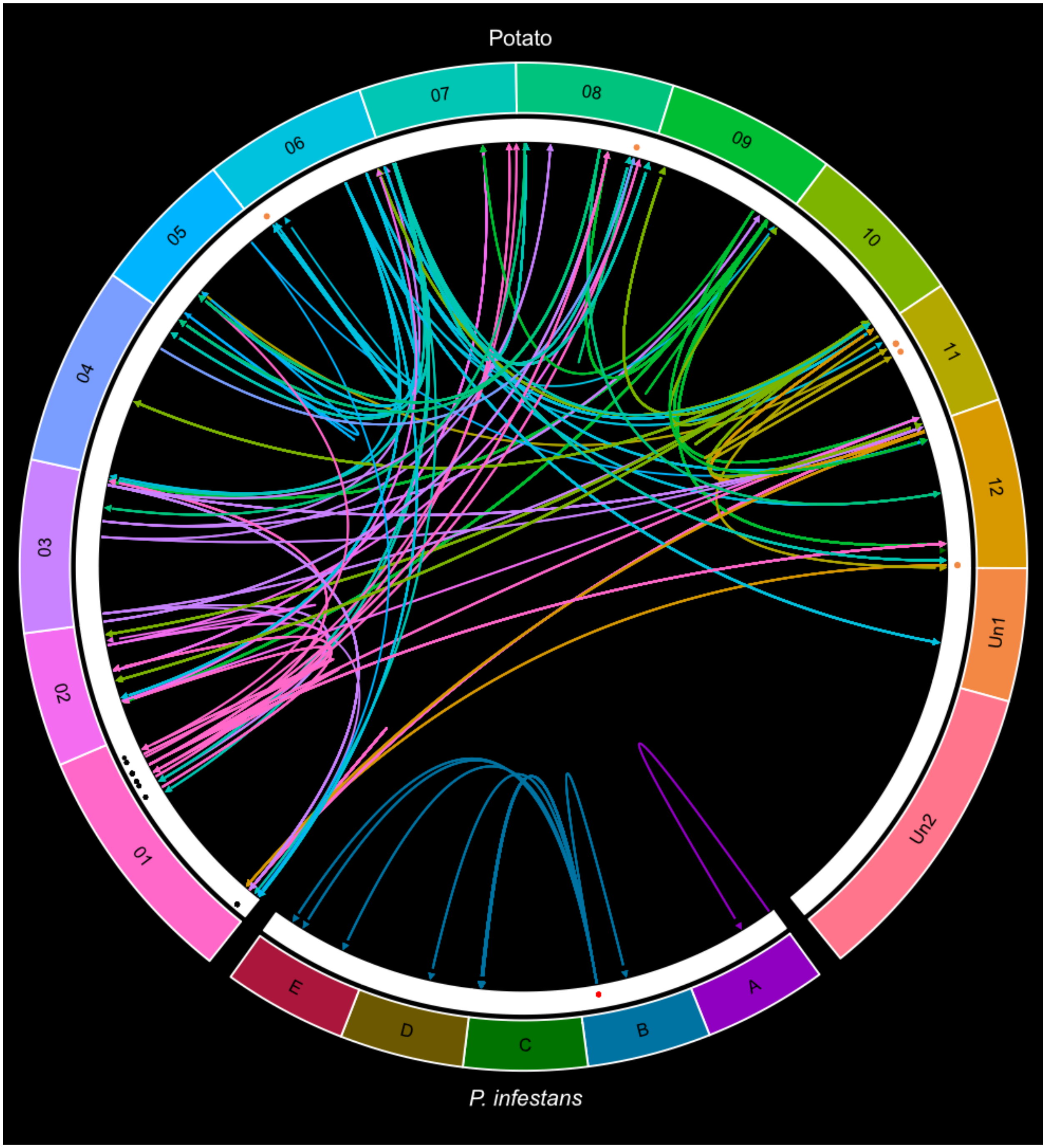

Figure S10 Precursor and target sites of sRNAs in NFA decreased datasets. Organized chromosome vice (potato) or as groups of supercontigs (A-E) for *P. infestans*. Un1 and Un2 are the unanchored sequences from potato genome version 4.03 and 4.04, respectively. The arrows represent the connection between each precursor and target site (arrowhead) and are colored according to the precursor chromosome or region. Colored dots in the white margin represent the following category of precursor sRNA loci: phasiRNAs (orange), TE (red), miRNAs (black).

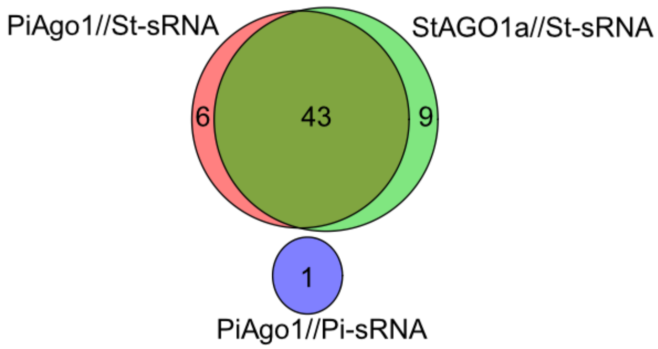

Figure S11 Number of true target genes after CNN filtration in the resistance gene version of the PiAgo1//St-sRNA, StAGO1a//St-sRNA and PiAgo1//Pi-sRNA datasets.

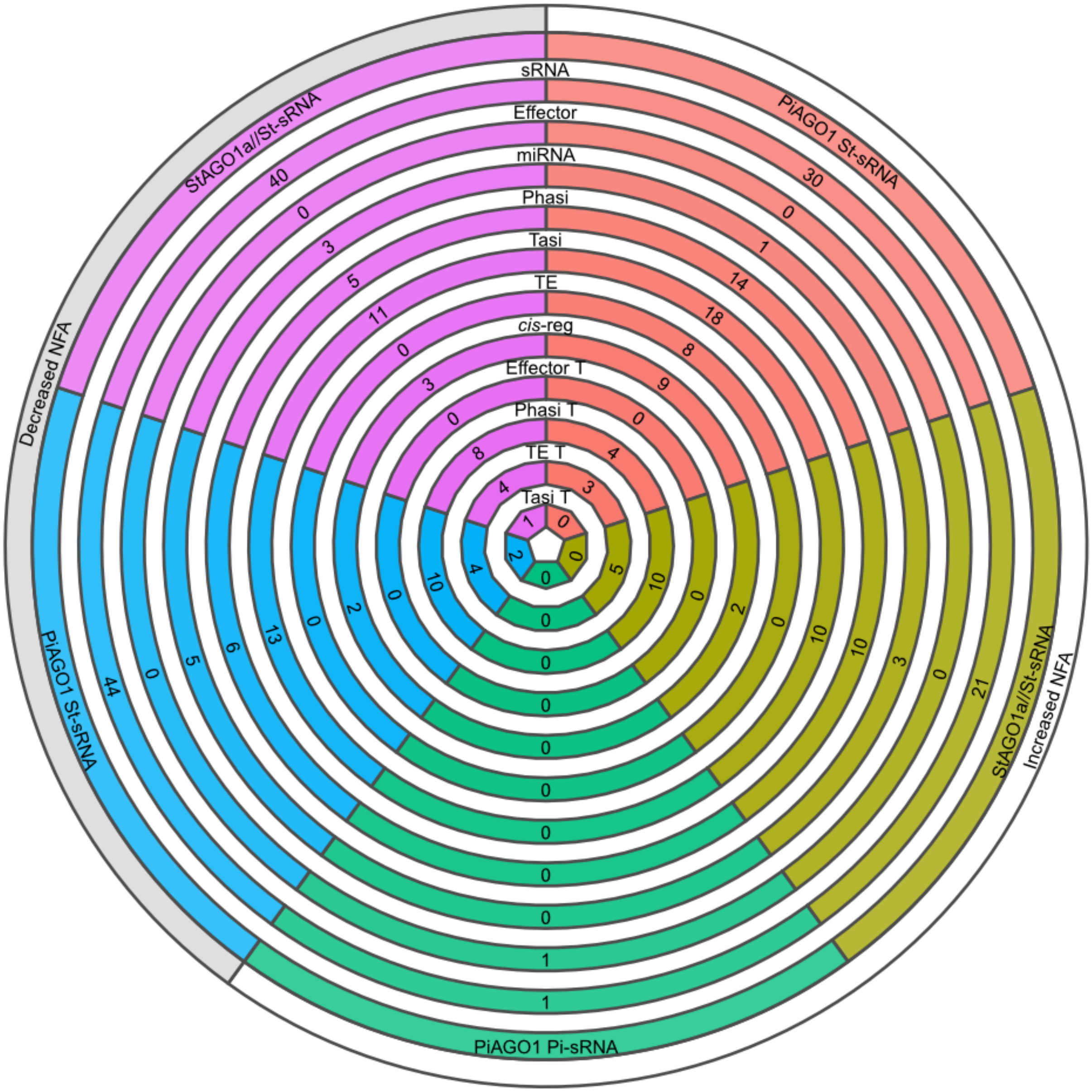

Figure S12 Precursor and target site summary (number of genes excluded) of the *R* gene dataset. White ring increased NFA. Grey ring decreased NFA. Subsequent rings towards the center corresponds to different materials, RNA classes and number of sRNAs related to each class. All classes denoted ¨T¨ in the end implies that the class corresponds to the target site. TE (transposons and repeats), *cis*-reg (*cis*-regulatory sRNAs). Phasi (phasiRNAs), tasi (tasiRNAs) are according to earlier suggestion [3].

A

B

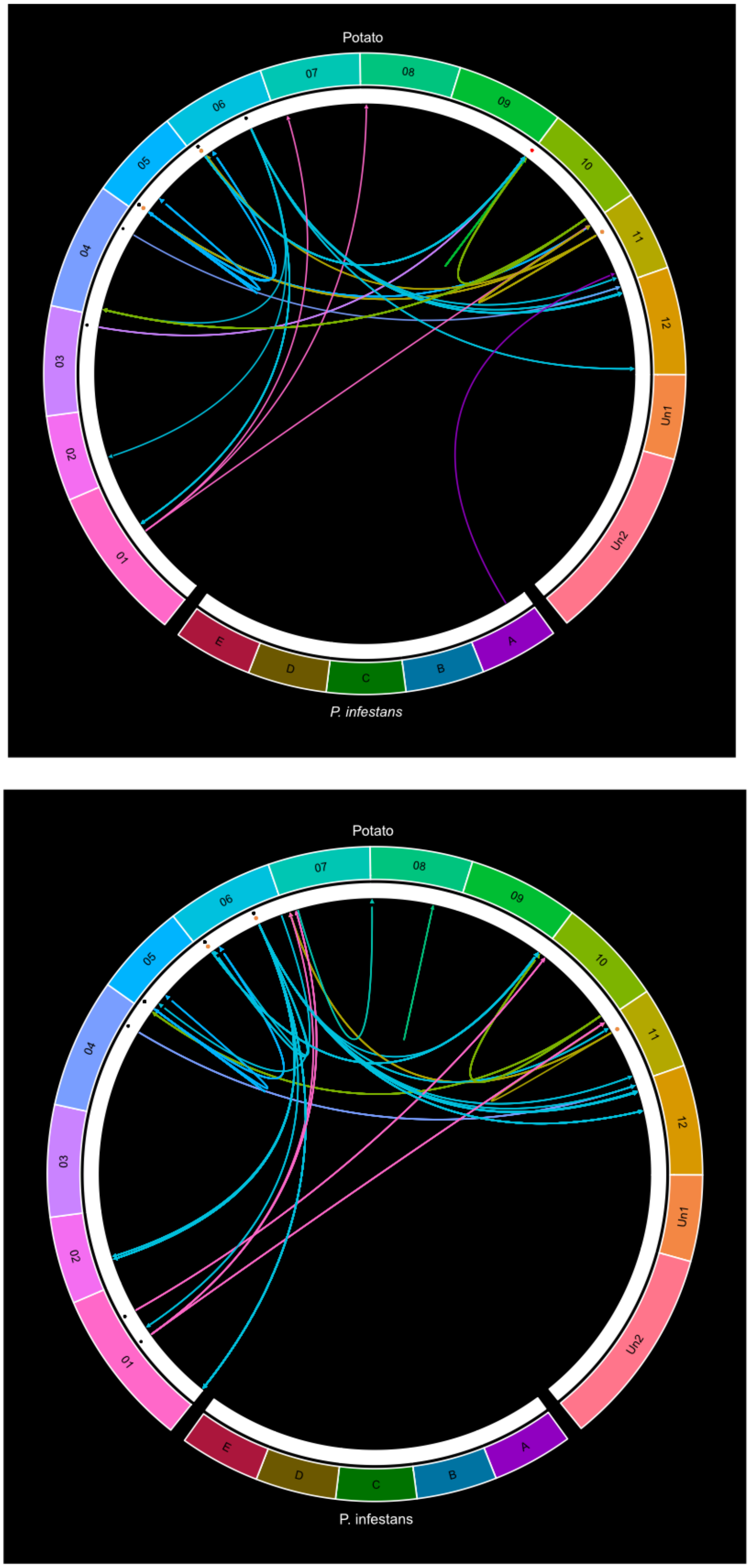

Figure S13 Precursor and target sites of endo- and exogenous sRNAs in the resistance gene datasets. Organized chromosome vice (potato) or as groups of supercontigs (A-E) for *P. infestans*. Un1 and Un2 are the unanchored sequences from potato genome version 4.03 and 4.04, respectively. (**A**) Resistance genes with increased NFA. (**B**) Resistance genes with decreased NFA. The arrows represent the connection between each precursor and target site (arrowhead) and are colored according to the precursor chromosome or region. Colored dots in the white margin represent the following category of the precursor sRNA loci: phasiRNA (light blue), TEs (red), miRNAs (black).

Table S1. Number of reads after quality filtration in all 15 datasets included in the analysis. Beside degradome sRNAs, reads from sRNA datasets from the following sequenced materials were used: (StAGO1a), transgenic potato harboring *StAGO1a-GFP*; (PiAgo1), transgenic *P. infestans* harboring *PiAgo1-GFP*. Wild-type (Wt) strain 88069 was used as mycelia and for potato leaf inoculation. Additional sRNA-sequences are named "background".

**Degradome**	
**Datasets**	**Number of reads**
Mycelia (*P. infestans* PiAgo1)	51 448 540
Mycelia (*P. infestans* Wt)	50 975 799
Leaves (H_2O inoculated)	67 110 295
Leaves (*P. infestans* PiAgo1 inoculated)	62 153 078
Leaves (*P. infestans* Wt inoculated)	58 927 635
**sRNA seq. from transgenic potato StAGO1a**	
St-sRNA (Leaves, H_2O inoculated)	7 064 140
St-sRNA (Leaves *P. infestans* Wt inoculated)	5 496 651
Pi-sRNA (Leaves, *P. infestans*, Wt inoculated)	287 592
**sRNA seq. from transgenic *P. infestans* PiAgo1**	
St-sRNA (Leaves, *P. infestans* PiAgo1 inoculated)	1 508 573
Pi-sRNA (Mycelia, *P. infestans*, PiAgo1)	16 007 106
Pi-sRNA (Leaves, *P. infestans* PiAgo1 inoculated)	5 500 958
**"Background" sRNA sequencing**	
St-sRNA (Leaves, H_2O inoculated)	2 164 699
St-sRNA (Leaves *P. infestans* Wt, inoculated)	2 471 282
Pi-sRNA (Mycelia, *P. infestans* Wt)	5 648 317
Pi-sRNA (Leaves *P. infestans,* Wt inoculated)	162 655

Table S2. Comparison datasets (CDs) were created by comparison between infection datasets and non-infection datasets. Each CD is presented in two versions, increased normalized fragment abundance (NFA) dataset and decreased NFA dataset. Any target in the technical created fallacious datasets (red text) were eliminated from the corresponding infection dataset, hence datasets with technical created fallacious controls only exist as increased NFA dataset.

**Comparison datasets**	**Target genome**	**Infected datasets**	**Non-infected datasets**
StAGO1a//St-sRNA	Potato	St-sRNA cleavages during infection	St-sRNA cleavages during inoculation with water
StAGO1a//Pi-sRNA	Potato	Pi-sRNA cleavages during infection	Pi-sRNA cleavages during inoculation with water
StAGO1a//St-sRNA	*P. infestans*	St-sRNA cleavages during infection	St-sRNA cleavages in mycelia
StAGO1a//Pi-sRNA	*P. infestans*	Pi-sRNA cleavages during infection	Pi-sRNA cleavages in mycelia
PiAgo1//St-sRNA	Potato	St-sRNA cleavages during infection	St-sRNA cleavages during inoculation with water
PiAgo1//Pi-sRNA	Potato	Pi-sRNA cleavages during infection	Pi-sRNA cleavages during inoculation with water
PiAgo1//St-sRNA	*P. infestans*	St-sRNA cleavages during infection	St-sRNA cleavages in mycelia
PiAgo1//Pi-sRNA	*P. infestans*	Pi-sRNA cleavages during infection	Pi-sRNA cleavages in mycelia
BG//St-sRNA	Potato	St-sRNA cleavages during infection	St-sRNA cleavages during inoculation with water
BG// Pi-sRNA	Potato	Pi-sRNA cleavages during infection	Pi-sRNA cleavages during inoculation with water
BG//St-sRNA	*P. infestans*	St-sRNA cleavages during infection	St-sRNA cleavages in mycelia
BG// Pi-sRNA	*P. infestans*	Pi-sRNA cleavages during infection	Pi-sRNA cleavages in mycelia

Table S3. Summary of final model visualizing the shape and number of parameters (param #) for each layer of the model.

Model: "sequential"

Loss function: "categorical_crossentropy"

Layer (type)	Output Shape	Param #
conv2d (Conv2D)	(None, 28, 28, 32)	896
spatial_dropout2d (SpatialDropout2D)	(None, 28, 28, 32)	0
batch_normalization (BatchNormalization)	(None, 28, 28, 32)	128
conv2d_1 (Conv2D)	(None, 28, 28, 64)	18496
max_pooling2d (MaxPooling2D)	(None, 14, 14, 64)	0
spatial_dropout2d_1 (SpatialDropout2D)	(None, 14, 14, 64)	0
batch_normalization_1 (BatchNormalization)	(None, 14, 14, 64)	256
flatten (Flatten)	(None, 12544)	0
dropout (Dropout)	(None, 12544)	0
dense (Dense)	(None, 64)	802880
dropout_1 (Dropout)	(None, 64)	0
dense_1 (Dense)	(None, 128)	8320
dropout_2 (Dropout)	(None, 128)	0
dense_2 (Dense)	(None, 3)	387

Total params: 831,363

Trainable params: 831,171

Non-trainable params: 192

Table S4. Tunable parameters in the Bayesian optimization. Minimum (Min) and maximum (Max) limits for hyperparameters, and settings of the maximum scoring model of the 92nd iteration (Final) are outlined.

**Hyperparameter**	**Min**	**Max**	**Final**
Epochs	100	300	237
Batch size	32	128	32
Dropout	0	0.3	0.014
Validation split	0.1	0.4	0.1
Convolutional loop iterations	0	4	1
Dense loop iterations	0	4	2
Max pooling iterations	1	2	1

Table S5. miRNA-mRNA cleavage data from earlier studies detected in the present analysis.

miRNA	miRNA length	miRNA sequence	Target transcript	CS	Target potato gene	Former reports
miR156d-5p	20	UUGACAGAAGAUAGAGAGCA	PGSC0003DMT400076399	1610	*SPL*	[4,5]
miR159c-3p	21	UUUGGAUUGAAGGGAGCUCCA	PGSC0003DMT400058426	1127	*GAMYB-like*	[6]
miR160a-5p	21	UGCCUGGCUCCCUGUAUGCCA	PGSC0003DMT400020874	1762	*ARF*	[7,8]
miR160a-5p	21	UGCCUGGCUCCCUGUAUGCCA	PGSC0003DMT400055519	2024	*ARF16*	[7,8]
miR160a-5p	21	UGCCUGGCUCCCUGUAUGCCA	PGSC0003DMT400055520	330	*ARF16*	[7,8]
miR160a-5p	21	UGCCUGGCUCCCUGUAUGCCA	PGSC0003DMT400062489	1499	*ARF16*	[7,8]
miR164e-5p	21	UGGAGAAGCAGGGCACGUGCA	PGSC0003DMT400050262	945	*StNAC262*	[9]
miR166b	21	UCGGACCAGGCUUCAUUCCUC	PGSC0003DMT400030829	737	*PHAVOLUTA-like HD-ZIPIII protein*	[10,11,12]
miR166b	21	UCGGACCAGGCUUCAUUCCUC	PGSC0003DMT400054420	844	*PHAVOLUTA-like HD-ZIPIII protein*	[10,11,12]
miR166b	21	UCGGACCAGGCUUCAUUCCUC	PGSC0003DMT400054422	841	*PHAVOLUTA-like HD-ZIPIII protein*	[10,11,12]
miR166b	21	UCGGACCAGGCUUCAUUCCUC	PGSC0003DMT400054423	233	*PHAVOLUTA-like HD-ZIPIII protein*	[10,11,12]
miR166b	21	UCGGACCAGGCUUCAUUCCUC	PGSC0003DMT400074932	565	*HD-ZipIII protein 8*	[10,11,12]
miR166b	21	UCGGACCAGGCUUCAUUCCUC	PGSC0003DMT400074934	979	*HD-ZipIII protein 8*	[10,11,12]
miR403b	20	CUAGAUUCACGCACAAACUC	PGSC0003DMT400054667	2404	*Argonaute 3*	[13]
miR6024-3p	22	UUUUAGCAAGAGUUGUUUUCCC	PGSC0003DMT400005011	575	*Rx protein*	[14]

Table S6. Potato miRNA families.

miRNA family	No. of members
miR482	5
miR403	4
miR166	3
miR156	1
miR159	1
miR171	1
miR384	1
miR408	1
miR6023	1
miR6024	1
miR8036	1

Table S7. Precursor summary of sRNA targeting in potato and *P. infestans*. TE (transposons and repeats), *cis*-reg (*cis*-regulatory sRNAs), phasi (phasiRNAs), tasi (tasiRNAs) are according to earlier suggestion [3].

**Dataset**	**No. sRNA**	**Effector**	**miRNA**	**Phasi**	**Tasi**	**TE**	***cis*-reg**
Endogenous St-sRNAs	566	0	20	115	39	11	64
Endogenous Pi-sRNAs	222	14	0	0	0	76	4
Translocating St-sRNAs	14	0	1	0	1	0	0
Translocating Pi-sRNAs	91	12	0	0	0	12	0

Table S8. Number of *PHAS* loci per nucleotide length of each individual locus.
One locus produced both 25 and 27 nt sRNA, hence each nt length was assigned 0.5 for that loci.

Nucleotides	No. phasiRNA clusters
18	3
19	5
20	1
21	96
22	2
23	4
25	2.5
27	0. 5

Table S9. Target genes with increased NFA in the *R* gene dataset. "No annotation" is assigned with #N/A.

Potato genes	Type	Predicted function
**PiAGO1//St-sRNA and StAGO1a//St-sRNA**		
PGSC0003DMG400002357	TIR-NB-LRR	Bacterial spot disease resistance protein 4
PGSC0003DMG400002426	TIR-NB-LRR	Resistance gene
PGSC0003DMG400007743b	NB-LRR	#N/A
PGSC0003DMG400007872	NB-LRR	NBS-LRR protein
PGSC0003DMG400009633	TIR-NB-LRR	N protein
PGSC0003DMG400009686	TIR-NB-LRR	Bacterial spot disease resistance protein 4
PGSC0003DMG400011524	NBARC	EDNR2GH3 protein
PGSC0003DMG400011898	NB-LRR	Tospovirus resistance protein B
PGSC0003DMG400015681	TIR-NB-LRR	Resistance gene
PGSC0003DMG400018429	TIR-NB-LRR	Bacterial spot disease resistance protein 4
PGSC0003DMG400024055	TIR-NB-LRR	Nematode resistance
PGSC0003DMG400026433	TIR-NB-LRR	ATP binding protein
PGSC0003DMG402032547	CC-NB-LRR	HJTR2GH1 protein
PGSC0003DMG404026432	TIR-NB-LRR	TIR-NBS-LRR resistance protein
RDC0001NLR0225	NB-LRR	#N/A
RDC0001NLR0322	NB-LRR	#N/A
**StAGO1a//St-sRNA**		
PGSC0003DMG400004295	NB-LRR	NBS-LRR protein
PGSC0003DMG400007870	NB-LRR	NBS-LRR protein
PGSC0003DMG400011529	CC-NB-LRR	R2
PGSC0003DMG400013091	NB-LRR	Disease resistance protein Gpa2
PGSC0003DMG400016600	CC-NB-LRR	Tospovirus resistance protein E
PGSC0003DMG400016983	TIR-NB-LRR	Bacterial spot disease resistance protein 4
PGSC0003DMG400018461	TIR-NB-LRR	Resistance gene
PGSC0003DMG400019627	NB-LRR	Disease resistance protein Gpa2
PGSC0003DMG400024273	NB-LRR	Resistance protein PSH-RGH6
PGSC0003DMG400029415	TIR-NB-LRR	Nematode resistance protein
PGSC0003DMG400033160	TIR-NBARC	TMV resistance protein N
PGSC0003DMG401015682	TIR-NB-LRR	NL25
PGSC0003DMG402016981	TIR-NB-LRR	Bacterial spot disease resistance protein 4
RDC0001NLR0173	NB-LRR	#N/A
RDC0001NLR0224	NB-LRR	#N/A
RDC0001NLR0256	NB-LRR	#N/A

**PiAGO1//St-sRNA**		
PGSC0003DMG400006570	NB-LRR	Tospovirus resistance protein C
PGSC0003DMG400007743a	TIR-NB-LRR	#N/A
PGSC0003DMG400018428	TIR-NB-LRR	Bacterial spot disease resistance protein 4
RDC0001NLR0220	NB-LRR	#N/A
**PiAGO1//Pi-sRNA**		
PGSC0003DMG400033334	TIR-NB-LRR	Bacterial spot disease resistance protein 4

Table S10. Target genes with decreased NFA in the *R* gene dataset. No annotation is assigned with #N/A.

**PiAGO1//St-sRNA and StAGO1//St-sRNA**		
**Potato genes**	**Type**	**Predicted function**
PGSC0003DMG400002426	TIR-NB-LRR	Resistance gene
PGSC0003DMG400004295	NB-LRR	NBS-LRR protein
PGSC0003DMG400013090	TIR-NB-LRR	Leucine-rich repeat-containing protein
PGSC0003DMG400013091	NB-LRR	Disease resistance protein Gpa2
PGSC0003DMG400018429	TIR-NB-LRR	Bacterial spot disease resistance protein 4
PGSC0003DMG400021477	NB-LRR	Cc-nbs-lrr resistance protein
PGSC0003DMG400021887	TIR-NB-LRR	Bacterial spot disease resistance protein 4
PGSC0003DMG400022785	NB-LRR	Resistance protein PSH-RGH6
PGSC0003DMG400024055	TIR-NB-LRR	Nematode resistance
PGSC0003DMG401009819	NB-LRR	Resistance protein PSH-RGH6
PGSC0003DMG402009818	NB-LRR	RGC1
RDC0001NLR0001	NB-LRR	#N/A
RDC0001NLR0298	NB-LRR	#N/A
PGSC0003DMG400001981	NB-LRR	NBS-LRR protein
PGSC0003DMG400002357	TIR-NB-LRR	Bacterial spot disease resistance protein 4
PGSC0003DMG400006576	NB-LRR	Tospovirus resistance protein A
PGSC0003DMG400007872	NB-LRR	NBS-LRR protein
PGSC0003DMG400013490	NB-LRR	Prf
PGSC0003DMG400020580	TIR-NB-LRR	Tir-nbs-lrr resistance protein
PGSC0003DMG400021863	NB-LRR	Nucleotide binding site-leucine rich repeat protein
PGSC0003DMG400024273	NB-LRR	Resistance protein PSH-RGH6
PGSC0003DMG401026432	TIR-NB-LRR	ATP binding protein
PGSC0003DMG401030700	TIR-NB-LRR	Resistance gene
PGSC0003DMG402002428	TIR-NB-LRR	NL25
PGSC0003DMG402016981	TIR-NB-LRR	Bacterial spot disease resistance protein 4
RDC0001NLR0076	NB-LRR	#N/A
RDC0001NLR0138	NB-LRR	#N/A
RDC0001NLR0173	NB-LRR	#N/A
RDC0001NLR0178	NB-LRR	#N/A
RDC0001NLR0225	NB-LRR	#N/A
**StAGO1a//St-sRNA**		
PGSC0003DMG400007385	CC-NB-LRR	CC-NB-LRR protein
PGSC0003DMG400007743b	NB-LRR	#N/A
PGSC0003DMG400026433	TIR-NB-LRR	ATP binding protein

PiAGO1//St-sRNA		
PGSC0003DMG401020581	TIR-NBARC	TIR-NBS-LRR resistance protein
RDC0001NLR0223	NB-LRR	#N/A
RDC0001NLR0224	NB-LRR	#N/A
PGSC0003DMG400019627	NB-LRR	Disease resistance protein Gpa2
PGSC0003DMG401015682	TIR-NB-LRR	NL25

Dataset information

Column description

genesT - Target gene transcript

cat – PAREsnip2 cleavage category

posT – Target position in the transcript

NFA – Normalized Fragment Abundance

NSRA – Normalized Short Read Abundance

NFA_C - Normalized Fragment Abundance of the related control sample

NSRA_C - Normalized Short Read Abundance of the related control sample

sRNA – small RNA

pos – precursor chromosome position of the sRNA

chrom - precursor chromosome

genomePos - precursor genome position of the sRNA

phasi – if phasiRNA, cluster annotation

origGene – original gene

origFunc- function of original gene

TE – If transposable element, what type

mirBase – If resemblance with mature miRNA in mirBase, what miRNA

Sm.Wa – Smith Waterman value of resemblance between the mirBase hit and the sRNA

Tasi – If coming from predicted TAS precursor

genomePosT - Target genome position of the sRNA

chromT – Target chromosome

phasT – If targeting phas loci transcript

TET – If targeting transposable element

genesTfunc – Function of the genes

TasiT - If targeting a TAS loci

cis-reg – If same precursor gene as target gene

Duplex – Cleavage site duplex (sRNA and target site nt)

genesTG - Target gene

Dataset 1. Precursor and target site information of endo- and exogenous sRNAs in the NFA increased datasets

Dataset 2. Precursor and target site information of endo- and exogenous sRNAs in the NFA decreased datasets

References

1. Haas, B.J.; Kamoun, S.; Zody, M.C.; Jiang, R.H.Y.; Handsaker, R.E.; Cano, L.M.; Grabherr, M.; Kodira, C.D.; Raffaele, S.; Torto-Alalibo, T.; et al. Genome sequence and analysis of the Irish potato famine pathogen *Phytophthora infestans*. *Nature* **2009**, 461, 393–398.
2. Hardigan, M.A.; Crisovan, E.; Hamilton, J.P.; Kim, J.; Laimbeer, P.; Leisner, C.P.; Manrique-Carpintero, N.C.; Newton, L.; Pham, G.M.; Vaillancourt, B.; et al. Genome reduction uncovers a large dispensable genome and adaptive role for copy number variation in asexually propagated *Solanum tuberosum*. *Plant Cell* **2016**, 28, 388–405.
3. Axtell, M.J. Classification and comparison of small RNAs from plants. *Ann. Rev. Plant Biol.* **2013**, 64, 137–159.
4. Wu, G.; Poethig, R.S. 2006. Temporal regulation of shoot development in *Arabidopsis thaliana* by *miR156* and its target *SPL3*. *Develop*. **2006**, 133, 3539–3547.
5. Xie, K.; Shen, J.; Hou, X.; Yao, J.; Li, X.; Xiao, J.; Xiong. L. Gradual increase of miR156 regulates temporal expression changes of numerous genes during leaf development in rice. *Plant Physiol.* **2012**, 158,1382–1394.
6. Alonso-Peral, M.M.; Li, J.; Li, Y.; Allen, R.S.; Schnippenkoetter, W.; Ohms, S.; White, R.G.; Milla, A.A. The microRNA159-regulated *GAMYB-like* genes inhibit growth and promote programmed cell death in Arabidopsis. *Plant Physiol.* **2010**, 154,757–771.
7. Mallory, A.C.; Bartel, D.P.; Bartel, B. MicroRNA-directed regulation of *Arabidopsis* AUXIN RESPONSE FACTOR17 is essential for proper development and modulates expression of early auxin response genes. *Plant Cell* **2005**,17,1360–1375.
8. Liu, P-P.; Montgomery, T.A.; Fahlgren, N.; Kasschau, K.D.; Nonogaki, H.; Carrington, J.C. Repression of AUXIN RESPONSE FACTOR10 by microRNA160 is critical for seed germination and post-germination stages. *Plant J.* **2007**, 52, 133–146.
9. Zhang, L.; Yao, L.; Zhang, N.; Yang, J; Zhu, X.; Tang, X: Calderón-Urrea, A.; Si, H. Lateral root development in potato is mediated by Stu-mi164 regulation of NAC transcription factor. *Front. Plant Sci.* **2018**, 9, 383.
10. Kim, J.; Jung, J-H.; Reyes, J.L.; Kim, Y-S.; Kim, S-Y.; Chung, K-S.; Kim, J.A.; Lee, M.; Lee, Y.; Narry Kim V.; et al. microRNA-directed cleavage of *ATHB15* mRNA regulates vascular development in Arabidopsis inflorescence stems. *Plant J.* **2005**, 42, 84–94.
11. Zhong, R.; Ye, Z-H. Regulation of *HD-ZIP III* genes by microRNA 165. *Plant Signal Behav.* **2007;** 2:351–353.
12. Jung, J-H.; Park, C-M. *MIR166/165* genes exhibit dynamic expression patterns in regulating shoot apical meristem and floral development in *Arabidopsis*. *Planta* **2007**, 225,1327–1338.
13. Zhang, C.; Xian, Z.; Huang, W.; Li, Z. Evidence for the biological function of miR403 in tomato development. *Scientia Horticult*. **2015**, 197, 619–626.
14. Li, F.; Pignatta, D.; Bendix, C.; Brunkard, J.O.; Cohn, M.M.; Tung, J.; Sun, H.; Kumar, P.; Baker, B. microRNA regulation of plant innate immune receptors. *Proc. Natl. Acad. Sci. USA* **2012**, 109, 1790–1795.
